# Establishment of tetracycline-regulated bimolecular fluorescence complementation assay to detect protein-protein interactions in *Candida albicans*

**DOI:** 10.1038/s41598-020-59891-7

**Published:** 2020-02-19

**Authors:** Wei-Chung Lai, H. Sunny Sun, Jia-Ching Shieh

**Affiliations:** 1 Department of Biomedical Sciences, Chung Shan Medical University, Taichung, Taiwan, ROC; 2 Institute of Molecular Medicine, College of Medicine, National Cheng Kung University, Tainan, Taiwan, R. O. C.; 3 Department of Medical Research, Chung Shan Medical University Hospital, Taichung, Taiwan, R. O. C.; 4 Immunology Research Centre, Chung Shan Medical University, Taichung, Taiwan, R. O. C.; 5 Present Address: Institute of Molecular Medicine, National Cheng Kung University Medical College, Tainan, Taiwan, R. O. C.

**Keywords:** Expression systems, Fungal genetics

## Abstract

To visualize protein-protein interactions in *Candida albicans* with the bimolecular fluorescence complementation (BiFC) approach, we created a Tet-on system with the plasmids pWTN1 and pWTN2. Both plasmids bear a hygromycin B-resistant marker (*CaHygB*) that is compatible with the original Tet-on plasmid pNIM1, which carries a nourseothricin-resistant marker (*CaSAT1*). By using GFPmut2 and mCherry as reporters, we found that the two complementary Tet-on plasmids act synergistically in *C. albicans* with doxycycline in a dose-dependent manner and that expression of the fusion proteins, *Ca*Cdc11-GFPmut2 and mCherry-*Ca*Cdc10, derived from this system, is septum targeted. Furthermore, to allow detection of protein-protein interactions with the reassembly of a split fluorescent protein, we incorporated mCherry into our system. We generated pWTN1-RN and pNIM1-RC, which express the N-terminus (amino acids 1–159) and C-terminus (amino acids 160–237) of mCherry, respectively. To verify BiFC with mCherry, we created the pWTN1-CDC42-RN (or pWTN1-RN-CDC42) and pNIM1-RC-RDI1 plasmids. *C. albicans* cells containing these plasmids treated with doxycycline co-expressed the N- and C-terminal fragments of mCherry either N-terminally or C-terminally fused with *Ca*Cdc42 and *Ca*Rdi1, respectively, and the *Ca*Cdc42-*Ca*Rdi1 interaction reconstituted a functional form of mCherry. The establishment of this Tet-on-based BiFC system in *C. albicans* should facilitate the exploration of protein-protein interactions under a variety of conditions.

## Introduction

Protein-protein interactions (PPIs) are a vital mechanism used to mediate a variety of cellular functions in many physiological environments. In the past, several biochemical strategies for the identification and characterization of these PPIs have been developed, such as affinity chromatography, co-immunoprecipitation, cross-linking and the pull-down assay^1^. In addition to biochemical methods, protein-fragment complementation methods, including the yeast two-hybrid method^2^, the split-ubiquitin system^3^ and bimolecular fluorescence complementation (BiFC)^4^, allow the direct analysis of protein interactions *in vivo*. BiFC invol ves the reassembly of a fluorescent complex through two non-fluorescent fragments fused to partners that interact^5^. PPIs in cells can be observed easily using fluorescent microscopy. The detection of PPIs through Förster resonance energy transfer (FRET), like BiFC, is another approach for the visualization of PPIs in living cells that uses two fluorophores, such as cyan and yellow fluorescent proteins (CFP and YFP), linked to the proteins of interest^6^. In FRET, light energy emitted by the excitation of a CFP fusion protein that is transferred to and excites a YFP fusion protein upon association of interacting fusion proteins^7^. The benefit of FRET over BiFC is that FRET allows the detection of the dynamic PPIs owing to its reversibility. However, BiFC allows the direct visualization of PPIs to be irreversible in living cells under a fluorescent microscope, eliminating cellular fixation. BiFC has been widely used in many model organisms from yeasts to mammals and plants^8–11^ to achieve the genome-wide screening for unknown interactions^12,13^, which cannot be accomplished with FRET.

*Candida albicans*, an opportunistic fungal pathogen that serves as a commensal on the mucosal surfaces of the gastrointestinal tract, is capable of developing pathogenesis in immunocompromised patients. Morphological transitions to adapt to environmental changes are related to a series of cellular processes that mediate blastopore formation as well as yeast, pseudohyphal or hyphal growth, which is closely linked to the virulence of *C. albicans*. Due to the incomplete sexual cycles and diploidy of *C. albicans* in nature, many genetic tools have been developed to study its cell biology^14,15^. The tetracycline-inducible gene expression system, which is composed of reverse tetracycline transactivator (rtTA) and a tetracycline response element, is a useful tool in *C. albicans* that allows gene expression to be completely dependent on the presence of tetracycline or its structural analogue doxycycline^16,17^. Introduction of dominant selectable markers to replace auxotrophic selectable markers is a desirable approach to facilitate the functional study of a gene in *C. albicans* without interference from nutrient metabolism^18–20^.

In this study, we improved the Tet-on system with the addition of two dominant selectable markers, *CaHygB*^21^ and *CaSAT1*, for gene manipulation in the wild-type SC5314 strain through integration of the *CaADH1* and *CaTDH3* loci. Next, we constructed *Ca*Cdc11-GFPmut2 and mCherry-*Ca*Cdc10 fusion proteins and verified their co-localization to the septin ring structure. Finally, we established an mCherry-based BiFC assay in *C. albicans* to detect PPIs using *Ca*Cdc42 and *Ca*Rdi1, which are involved in polarized growth^8,22,23^. The BiFC system is an effective visual assay to detect PPIs in wild-type *C. albicans* strains.

## Methods

### Strains and growth conditions

*C. albicans* SC5314^24^ was used as the wild-type strain in this study. The wild-type strain and all derivative strains listed in Table 1 were routinely grown in YPD (1% yeast extract, 2% peptone, and 2% glucose) medium adjusted to pH 5.4 supplemented with the appropriate antibiotics and on solid plates containing 2% agar. The antibiotics used were hygromycin B (1 mg/ml, Gold Biotechnology, USA) and nourseothricin (200 µg/ml, Werner Bioagents, Germany). Transformants were selected on YPD agar containing nourseothricin, hygromycin B or both. To induce expression of fluorescent proteins, strains were diluted in YPD containing doxycycline (40 µg/ml, D9891, Sigma) and grown overnight for 3 hours. The concentration of doxycycline was two-fold diluted from 40 to 0.1 µg/ml to test the effect of different doxycycline doses. For cellular morphology-specific growth, cells were grown in YPD overnight and diluted 1:10 in fresh YPD at 30 °C (for yeast cells) or YPD with 10% fetal bovine serum (FBS, HyClone) for growth at 37 °C (for hyphal cells) for 3 hours.

**Table 1:** *C. albicans* strains used in this study.

Strain	Parent	Genotype	Plasmids^a^	Protein expressed^a^	Reference
SC5314		Wild-type strain			(Gillum *et al*.^24^)
WCL101	SC5314	*ADH1/adh1*::*P*_*TET*_*-GFPmut2:SAT1*	pNIM1-NC	GFPmut2	This study
WCL102	SC5314	*TDH3/tdh3*::*P*_*TET*_*-GFPmut2:HygB*	pWTN1	GFPmut2	This study
WCL103	SC5314	*TDH3/tdh3*::*P*_*TET*_*-mCherry:HygB*	pWTN2	mCherry	This study
WCL104	WCL101	*ADH1/adh1*::*P*_*TET*_*-GFPmut2:SAT1 TDH3/tdh3*::*P*_*TET*_*-GFPmut2:HygB*	pWTN1 & pNIM1-NC	GFPmut2	This study
WCL105	WCL102	*ADH1/adh1*::*P*_*TET*_*-GFPmut2:SAT1 TDH3/tdh3*::*P*_*TET*_*-mCherry:HygB*	pWTN2 & pNIM1-NC	mCherry & GFPmut2	This study
WCL106	SC5314	*TDH3/tdh3*::*P*_*TET*_ *-CDC42-mCherry (1-159):HygB*	pWTN1-6H-CDC42-RN	Cdc42-RN	This study
WCL107	SC5314	*ADH1/adh1*::*P*_*TET*_ *-mCherry (160-237) -RDI1:SAT1*	pNIM1-RC-RDI1	RC-Rdi1	This study
WCL108	WCL106	*TDH3/tdh3*::*P*_*TET*_ *-CDC42-mCherry (1-159):HygB ADH1/adh1*::*P*_*TET*_ *-mCherry (160-237) -RDI1:SAT1*	pWTN1-6H-CDC42-RN & pNIM1-RC-RDI1	Cdc42-RN & RC-Rdi1	This study
WCL109	SC5314	*TDH3/tdh3*::*P*_*TET*_ *-mCherry (1-159):HygB*	pWTN1-RN	RN	This study
WCL110	SC5314	*ADH1/adh1*::*P*_*TET*_ *-mCherry (160-237):SAT1*	pNIM1-RC	RC	This study
WCL111	WCL109	*TDH3/tdh3*::*P*_*TET*_ *-mCherry (1-159):HygB ADH1/adh1*::*P*_*TET*_ *-mCherry (160-237):SAT1*	pWTN1-RN & pNIM1-RC	RN & RC	This study
WCL112	WCL109	*TDH3/tdh3*::*P*_*TET*_ *-mCherry (1-159):HygB ADH1/adh1*::*P*_*TET*_ *-mCherry (160-237) -RDI1:SAT1*	pWTN1-RN & pNIM1-RC-RDI1	RN & RC-Rdi1	This study
WCL113	WCL106	*TDH3/tdh3*::*P*_*TET*_ *-CDC42-mCheery (1-159):HygB ADH1/adh1*::*P*_*TET*_ *-mCherry (160-237):SAT1*	pWTN1-6H-CDC42-RN & pNIM1-RC	Cdc42-RN & RC	This study
WCL114	SC5314	*TDH3/tdh3*::*P*_*TET*_*-mCherry-CDC10:HygB*	pWTN2-mCherry-CDC10	mCherry-Cdc10	This study
WCL115	SC5314	*ADH1/adh1*::*P*_*TET*_*-CDC11-GFPmut2:SAT1*	pNIM1-CDC11-GFPmut2	Cdc11-GFPmut2	This study
WCL116	WCL114	*TDH3/tdh3*::*P*_*TET*_*-mCherry-CDC10:HygB ADH1/adh1*::*P*_*TET*_*-CDC11-GFPmut2:SAT1*	pWTN2-mCherry-CDC10 & pNIM1-CDC11-GFPmut2	mCherry-Cdc10 & Cdc11-GFPmut2	This study
WCL117	SC5314	*TDH3/tdh3::P*_*TET*_ *-mCherry (1-159)-CDC42:HygB*	pWTN1-RN-CDC42	RN-Cdc42	This study
WCL118	WCL117	*TDH3/tdh3::P*_*TET*_ *-mCherry (1-159)-CDC42:HygB ADH1/adh1::P*_*TET*_ *-mCherry (160-237) -RDI1:SAT1*	pWTN1-RN-CDC42 & pNIM1-RC-RDI1	RN-Cdc42 & RC-Rdi1	This study
WCL125	WCL117	*TDH3/tdh3::P*_*TET*_ *-mCherry (1-159)-CDC42:HygB ADH1/adh1::P*_*TET*_ *-mCherry (160-237):SAT1*	pWTN1-RN-CDC42 & pNIM1-RC	RN-Cdc42 & RC	This study

^a^RN indicated as mCherry (1–159) and RC indicated as mCherry (160–237).

### Construction of plasmids

All primers used in this study are listed in Table 2. Details of the construction of plasmids used in this study are described in the Supplemented Materials and Methods.

**Table 2:** Synthetic oligonucleotides used in this study.

Primer	Sequence^b^
TDH3p SacII F	ATTA***CCGCGG***GTTGCTCCTCGTCGACAA
TDH3p XbaI R	CGGG***TCTAGA***CATTGTTAATTAATTTGATTGTAAAG
MCS3 F	CTAGATTTAAATATGCATTGACCGCGGATTCAAGAGTCCAAGCT
MCS3 R	TGGACTCTTGAATCCGCGGTCAATGCATATTTAAAT
MCS4 F	TGCACCAGCTCCGGTACCACTAGTCATGTTTAAACCGG
MCS4 R	TCGACCGGTTTAAACATGACTAGTGGTACCGGAGCTGGTGCAGTAC
HygB BglII F	GCTT***AGATCT***CCTGCAGGTATAGTGCTTGCTGTTCGATA
HygB NsiI R	CGTT***ATGCAT***ACGCGTCATTTTATGATGGAATGAATGGG
TetO F	AAACTCGAGTTTACCACTCCCTATCAGTGATAGAGAAAAGTGAAAGTCGACATTGAGCT
TetO R	CAATGTCGACTTTCACTTTTCTCTATCACTGATAGGGAGTGGTAAACTCGAGTTT
miniOP4 SpeI F	GATT***ACTAGT***CATTATTTATATTTGTATGTGTGTAGGAGTT
miniOP4 XhoI R	AGGA***CTCGAG***CAAACTTCTTTTTTATTTTTCGCAATAT
TDH3t PmeI F2	AAAA***GTTTAAAC***ACCAAACTCGAAGGTGCTC
TDH3t KpnI R2	GATT***GGTACC***TCTGGTGGAGTAACCGTATT
GFPt XbaI F	TTT***TCTAGA***CCGGTCCGACATTTTATGATGGAATGAATGG
GFPt SpeI R	TTT***ACTAGT***GTCGACTCGAGATATCCAGCTA
mRFP NotI F	ATTT***GCGGCCGC***TCCGGAGTTTCAAAAGGTGA
mRFP AatII R	CATA***GACGTC***AGGCGCGCCTTTATATAATTCA
GS F	ATCAGATCTGGTGGAGGTG
GS R	GATCCACCTCCACCAGATCTGAT
mRFP N BamHI F	AACT***GGATCC***ATGGTTTCAAAAGGTGAAGAAGATAATATGGC
mRFP N NotI R	TCTA***GCGGCCGC***AGGCCTTTAATCTTCTGGATACATTCTTTCTGATGAAGC
mRFP C XhoI F	TCAA***CTCGAG***ATGGGTGCTTTAAAAGGTGAAATTAAACAAAGA
mRFP C BamHI R	TACT***GGATCC***TTTATATAATTCATCCATACCACCAGTTGA
CaGFP-N-MCS	AATT***GTCGACTCGAGATATC***CA***GCTAGCGGCCGC***GTGACCATGAGTAAGGGAGAAGAAC
CaGFP-C-MCS	CCG***TGATCA***TTATGC***AGGCCTAGATCTTAAG***CT***GACGTCCGGA***CCTTTGTATAGTTCATCCATGCC
CDC42 EcoRV F	CCTA***GATATC***CAAACTATAAAATGTGTTGTTGTCGGTG
CDC42 BglII R	GGGT***AGATCT***TAAAATAGTACACTTTTTCGATTTTTTAAT
RDI1 SpeI F	GATT***ACTAGT***TCCAATTCTAACGTTGATGATGAT
RDI1 StuI R	GCTT***AGGCCT***CTATTTAGTAATGGAAAAACTCCAAGG
RedN KpnI F	CCAA***GGTACCACTAGT***ATGGTTTCAAAAGGTGAAGAAGATAATATGGC
RedN EcoRV	CTTT***GATATC***ATCTTCTGGATACATTCTTTCTGATGAAGC
CaCDC42 BamHI F	ATAA***GGATCCGACGTC***ATGCAAACTATAAAATGTGTTGTTGTCG
CaCDC42 NotI R	ATAA***GCGGCCGCAGGCCT***CTATAAAATAGTACACTTTTTCGATTTTTTAAT
pNIM1 inte F	CATGTCAAAGGATTCAAC
pNIM1 inte R	GTATGGTGCCTATCTAAC
TDH3 F	TTTGGTTGCGTTAGTCCG

^b^Restriction enzyme sites included in primers are highlighted in bold italics.

### *C. albicans* transformation

Each of the strains was made by introducing the appropriate cassette via electroporation essentially as described^25^ with some previously described modifications^20^. The plasmid cassettes were excised as *Kpn*I/*SacI*I-digested fragments and extracted with a Gel/PCR DNA Fragments Extraction Kit (Geneaid, Taiwan). One microgram of linear DNA fragments was mixed with 50 μl of competent cells and electroporated in a 0.2 cm cuvette at 1.8 kV via the Gene Pulser Xcell™ system (Bio-Rad, USA). After electroporation, the competent cells were resuspended in 1 ml of ice-cold 1 M sorbitol and transferred to a collection tube. The cells were collected by centrifugation at 4000 r.p.m. and 4 °C and resuspended in 1 ml of YPD medium before incubation at 30 °C for 4 hours. Finally, the cells were plated onto YPD plates supplemented with the appropriate antibiotics and incubated at 30 °C for 1~2 days. The transformants were selected after screening by colony PCR (Supplemental Materials and Methods).

### Protein extraction and western blotting

Cells cultured after doxycycline induction were pelleted via centrifugation at 5000 r.p.m. and washed with PBS twice before protein extraction essentially as described previously^20^. The fluorescent proteins GFPmut2 and mCherry were detected with mouse anti-GFP antibody (11814460001, Roche, Germany) and rabbit anti-mCherry antibody (5993–100, BioVision, USA), respectively, according to the manufacturers’ instructions. β-Actin was used as a loading control and detected with rabbit anti-β-actin antibodies (GTX109639, GeneTex, USA). The PageRuler Prestained Protein Ladder (26616, Thermo Fisher Scientific, USA) was used to indicate the relevant sizes of the protein targets. Signals from the detected proteins were obtained with SuperSignal West Pico Chemiluminescent Substrate (34080, Thermo Fisher Scientific, USA), and images were captured with an ImageQuant LAS 4000 Mini system (GE Healthcare Life Sciences). To quantify the levels of GFPmut2 and mCherry on the blots, images were analysed in triplicate with ImageJ (National Institutes of Health, USA), and then the quantified GFPmut2 levels were normalized to those of β-actin to determine the fold change in expression. Differential expression data were assessed by one-way ANOVA with Bonferroni’s comparison test, and the data were plotted as bar charts by GraphPad Prism 6 (GraphPad Software, USA).

### Fluorescence microscopy

In general, *C. albicans* cells were grown at 30 °C overnight, diluted 1:10 into their respective media containing 40 μg/ml doxycycline, and grown for an additional 3 hours at a temperature specific for the yeast and filamentous forms. For the BiFC assay, the cells were grown in YPD medium with 40 μg/ml doxycycline at 30 °C for 6 hours. To prepare each slide, YPD containing 1% agarose was added to a depression slide, and the top of the agarose was flattened with another slide before hardening. Poly-L-lysine-coated slides were prepared by evenly spreading 10 μl of filter-sterilized 0.1% poly-L-lysine (P8920, Sigma) onto a degreased microscope glass slide with a coverslip, which was then air-dried. A few microlitres of cultured cells were gently added onto the surface of the slide and covered with a coverslip before gently pressing the cover slide against the slide and sealing the slides with clear nail polish. Differential interference contrast (DIC) and fluorescent images were captured by a ZEISS Axio Imager A2 microscope (Zeiss, Germany) with an X-Cite 120Q unit (Excelitas, Massachusetts, United States) with a 120 W mercury vapor short arc lamp. Images were captured with a Retiga EXi (Teledyne QImaging, Canada) and FITC and rhodamine filter sets with excitation/emission wavelengths of 488/509 nm for GFPmut2 and 587/610 nm for mCherry. A Plan Neofluar 100×/1.30 oil objective lens combined with a DIC prism slider was used to capture DIC images. The digital images were analysed by QCapture Pro 7 (Teledyne QImaging, Canada) and processed with ImageJ (National Institutes of Health, USA). To quantify the relative fluorescence intensity of the cells in the presence of doxycycline, randomly selected five fluorescence microscopy images from strains, WCL111, WCL112, WCL113, WCL108, WCL125, and WCL118, were analysed. The relative fluorescence intensity was formulated as the integrated intensity of the fluorescence subtracted the fluorescence of background measured by ImageJ. The fluorescence difference was calculated with one-way ANOVA with Bonferroni’s comparison test, and the data were plotted as bar charts by GraphPad Prism 6 (GraphPad Software, USA).

### Nucleotide sequence accession numbers

The sequence of the pWTN2 plasmid has been submitted to GenBank with accession number KP087797.

## Results

### Construction of an additional tetracycline-regulated gene expression system in *C. albicans* with the hygromycin B-resistance marker

**Figure 1:**
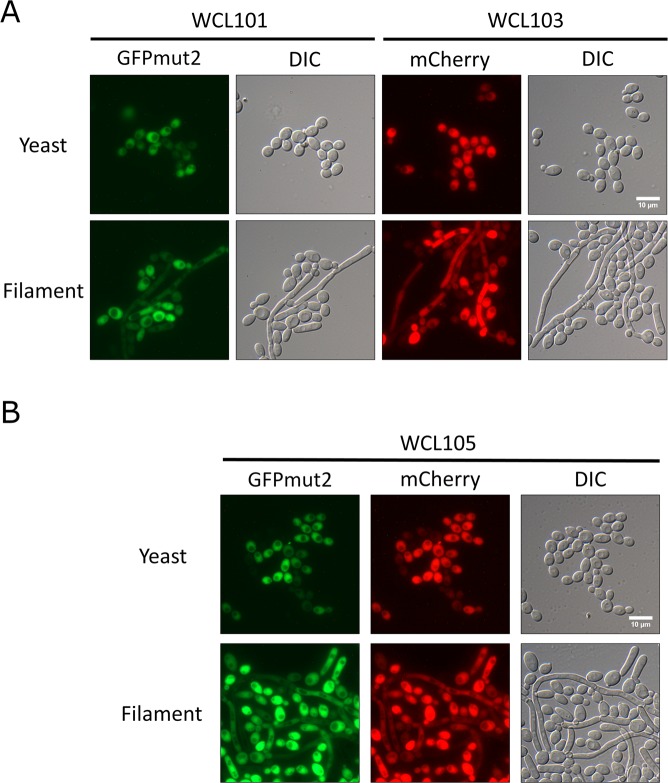
Expression of doxycycline-inducible fluorescent proteins in the yeast and filamentous forms of *C. albicans*. (**A**) The doxcycline-induced GFPmut2 and mCherry expression in yeast and filamentous forms of *C. albicans*. *C. albicans* cells from strains WCL101 and WCL103 were grown to exponential phase and for 3 hours after their transfer to either YPD at 30 °C (yeast) or YPD with 10% FBS at 37 °C (hyphae), both of which contain 40 μg/ml doxycycline. The fluorescent signals from the cells were observed under a fluorescence microscope. The bar indicates 10 μm. (**B**) Co-expression of GFPmut2 and mCherry induced by doxycycline in yeast and filamentous forms of *C. albicans*. GFPmut2 and mCherry were co-expressed by doxycycline in *C. albicans* cells from strain WCL105. The cells were grown in the conditions as in those used in (**A**). The fluorescent signals from this strain were detected by fluorescent microscopy. The bar indicates 10 μm.

A previous tetracycline-regulated gene expression system using the pNIM1 plasmid, also called the “Tet-on” system, in which the Tet-on cassette was integrated into the *CaADH1* locus, was established in *C. albicans*^16^. The tetracycline response element (*tet*O) fused to a minimal *CaOP4* promoter and the codon-optimized gene encoding reverse transactivator (rtTA) in the pNIM1 cassette constitute the key components of the Tet-on system. In this system, the nourseothricin-resistant gene *CaSAT1* serves as a dominant selection marker, and the homologous region of the *CaADH1* locus directs genomic integration of the cassette. That system is a convenient strategy for the expression of a target gene involved in molecular cloning and the subsequent transformation of a restriction enzyme-linearized cassette into wild-type *C. albicans*. The introduction of *Escherichia coli* hygromycin B phosphotransferase as an additional dominant selectable marker for hygromycin B resistance has been tested in *C. albicans*, and the simultaneous presence of hygromycin B and nourseothricin has also been confirmed to be compatible for the selection of *C. albicans*^21^. Based on these characteristics, we designed a Tet-on system with two plasmid vectors, pWTN1 and pWTN2, that is capable of gene expression under the control of tetracycline-regulated elements. This system contains a selectable hygromycin B-resistance marker that is flanked with homologous regions of the *CaTDH3* locus for its integration (Fig. S1). *CaTDH3* encodes glyceraldehyde-3-phosphate dehydrogenase (GAPDH), which is constitutively expressed in cells and carries out functions associated with the cell wall^26^; GAPDH is enriched at the stationary phase^27^, so *CaTDH3* was chosen as an alternative locus using its promoter to drive rtTA. To test the function of the new Tet-on system in *C. albicans*, pWTN1 and pWTN2 were linearized with *Sac*II and *Kpn*I and integrated into the *CaTDH3* locus of the wild-type *C. albicans* SC5314 by transformation, followed by hygromycin B selection; diagnostic colony PCR was then carried out for confirmation (Fig. S2), and the WCL102 and WCL103 strains, respectively, were eventually obtained. The WCL102 strain was capable of expressing GFPmut2, and the WCL103 strain was capable of expressing mCherry, which made the colonies pink in the visual spectrum (Fig. S3). Additionally, the pNIM1-NC plasmid, which was derived from pNIM1, was constructed through the addition of several restriction enzyme sites (Fig. S1). After linearization with *Kpn*I and *Sac*II, the pNIM1-NC plasmid was transformed and integrated at the *CaADH1* locus in *C. albicans* to obtain the WCL101 strain, which was capable of GFPmut2 expression, for use as a control. To assess the compatibility of nourseothricin and hygromycin B in the new system, the pNIM1-NC plasmid was further transformed into the WCL102 and WCL103 strains to obtain the WCL104 and WCL105 strains, respectively, each of which contained either two sets of Tet-on cassettes with two copies of the GFPmut2 coding sequence or two sets of Tet-on cassettes with one copy of the GFPmut2 coding sequence and one copy of the mCherry coding sequence. The doxycycline-inducible expression of GFPmut2 and mCherry in strains WCL101 and WCL103 under yeast and hyphal induction conditions was verified by fluorescence microscopy (Fig. 1A). Additionally, GFPmut2 and mCherry were simultaneously expressed in the WCL105 strain (Fig. 1B). These results indicate that the fluorescent proteins GFPmut2 and mCherry were expressed either individually or concurrently in both the yeast and hyphal forms by the newly constructed Tet-on system.

### Doxycycline regulates gene expression in a dose- and reverse transactivator-dependent manner

**Figure 2:**
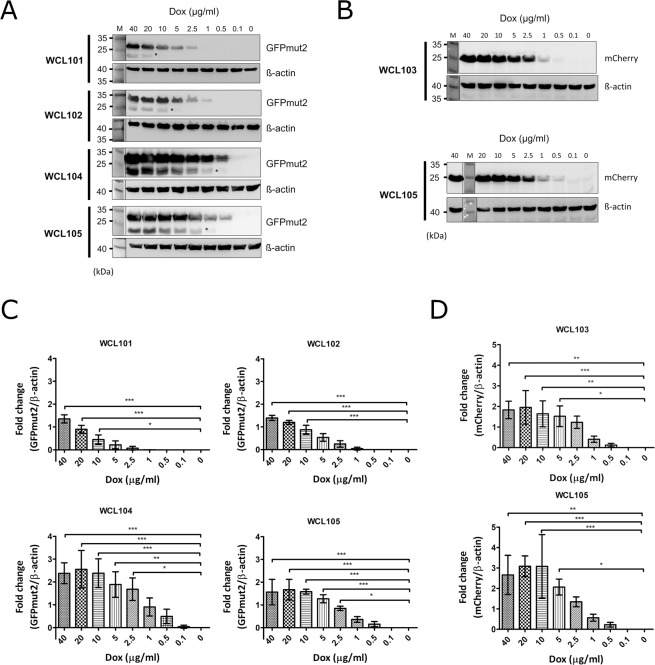
Quantification of the doxycycline-inducible expression level of fluorescent proteins in *C. albicans*. (**A**) GFPmut2 was used as an indicator to quantify expression under the Tet-on system in a dose-dependent manner. *C. albicans* cells from strains WCL101, WCL102, WCL104, and WCL105 were grown in YPD with the indicated concentration of doxycycline for 3 hours, followed by western blotting to analyse the amount of GFPmut2 in the cell lysates. β-Actin was used as an internal control. Asterisks indicate non-specific signals. (**B**) mCherry was used as another indicator to quantify expression under the Tet-on system in a dose-dependent manner. *C. albicans* cells from strains WCL103 and WCL105 were grown in YPD with the indicated concentration of doxycycline for 3 hours, and their lysates were subjected to western blotting to analyse the amount of mCherry in the cells. β-Actin was used as an internal control. (**C**) The change in doxycycline-inducible GFPmut2 expression. The GFPmut2 and β-actin signals on the blots shown in (**A**) were quantified with ImageJ. The bar charts show the fold change in GFPmut2 expression normalized to the relevant β-actin expression analysed with one-way ANOVA with Bonferroni’s comparison test. (**D**) The change in doxycycline-induced mCherry expression. The mCherry and β-actin signals on the blots shown in (**B**). The bar charts were obtained with the same formula used for (**C**). The numbers of asterisks show the significance of the difference between two values compared by statistical analysis.

In a previous study, we established an auxotrophic *C. albicans* strain carrying the Tet-on cassette of the pTET25M plasmid to confirm that the Tet-on system functions in a dose-dependent manner^17^. As there were two Tet-on systems in *C. albicans*, the conditions were more complicated than those used for yeast containing one Tet-on system. To determine the strength of gene expression under the two Tet-on systems, the expression of fluorescent proteins was induced in the WCL101, WCL102, WCL104, and WCL105 strains by doxycycline ranging in concentration from 40, 20, 10, 5, 2.5, 1, 0.5, 0.1 to 0 μg/ml and examined by western blotting. Comparison of GFPmut2 expression in the WCL101 and WCL102 strains, in which the individual Tet-on system was integrated at one allele of the *CaADH1* or *CaTDH3* loci, respectively, revealed that GFPmut2 was expressed in a dose-dependent manner in both strains (Fig. 2A). Additionally, the minimum dose of doxycycline required to induce GFPmut2 expression was different between the WCL101 and WCL102 strains. Through quantitative analysis of western blots performed in triplicate, we observed that the change in GFPmut2 expression was significant in WCL101 cells induced with 10 µg/ml doxycycline and WCL102 cells induced with 5 µg/ml doxycycline (Fig. 2A,C). These results suggested that the *CaTDH3* promoter is stronger than the *CaADH1* promoter and capable of driving more reverse transactivator (rtTA) expression, leading to the induction of GFPmut2 at a low dose of doxycycline. To better understand the relationship between the level of gene expression and the dose of doxycycline used to induce *C. albicans* with two Tet-on systems, we compared the amount of GFPmut2 expressed in the WCL101, WCL102, and WCL104 strains. The level of GFPmut2 expression in the WCL104 strain also correlated with the dose of doxycycline and plateaued with induction with 10 to 40 μg/ml doxycycline (Fig. 2A,C). However, the fold change in GFPmut2 expression in the WCL104 strain was as high as 2.5 in the presence of 40 μg/ml doxycycline, which was greater than the approximately 1.5-fold change in GFPmut2 expression in the WCL101 and WCL102 strains (Fig. 2C). The increased GFPmut2 level in the WCL104 strain indicates that the two Tet-on systems had a synergistic effect on target gene expression owing to the two copies of GFPmut2 and the constitutive expression of two rtTA genes. Moreover, the minimal dose of doxycycline required to induce GFPmut2 expression in the WCL104 and WCL105 strains decreased significantly down to 2.5 μg/ml doxycycline (Fig. 2C). A reasonable explanation for this observation is that the increased level of rtTA enriched its binding to doxycycline, leading to the induction of gene expression with a low dose of doxycycline. Although quantitative analysis showed the minimal (optimal) dose of doxycycline required for induction, it appeared that the Tet-on system in the WCL101, WCL102 and WCL105 strains was able to switch on slightly in the presence of doxycycline at 2.5, 1, 0.5, and 0.5 ug/ml, respectively (Fig. 2A). Additionally, to confirm the effects of rtTA on gene expression under two Tet-on systems in an alternative way, we compared the expression of mCherry by using western blotting of the WCL103 and WCL105 strains. As shown in Fig. 2B, mCherry was expressed in the WCL103 and WCL105 strains in a dose-dependent manner, similar to induced GFPmut2 expression in the WCL101 and WCL102 strains. Through quantification assays with statistical analyses (Fig. S4, Tables S1 and S2), the minimal (optimal) dose of doxycycline required to significantly induce mCherry was shown to be 5 μg/ml (Fig. 2D). However, doxycycline at 0.5 μg/ml induced the slight expression of mCherry (Fig. 2B). From these observations, we concluded that the level of rtTA driven by the stronger promoter mainly affects gene expression, differences in the doxycycline dose changed the level of the induced proteins, and the number of target gene copies had a minor effect on the level of the induced gene expression.

### Tracing the location of a structural protein in septin via two Tet-on systems

**Figure 3:**
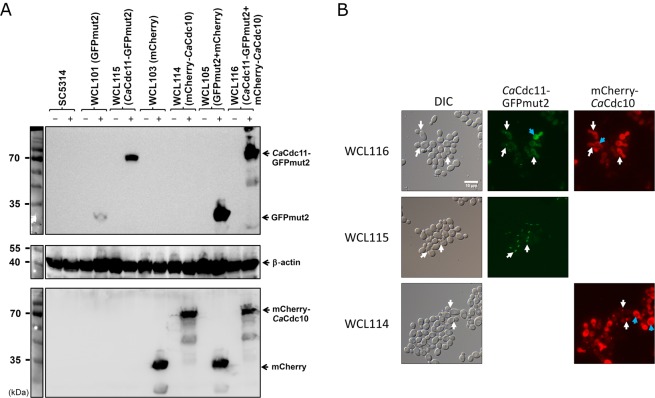
Expression of the doxycycline-induced *Ca*Cdc11-GFPmut2 and mCherry-*Ca*Cdc10 fusion proteins localized at the septum of *C. albicans*. (**A**) Examination of the expression of *Ca*Cdc11-GFPmut2 and mCherry-*Ca*Cdc10 in the presence of doxycycline by western blotting. *C. albicans* cells from strains WCL114, WCL115, and WCL116 were grown in YPD with (+) or without (−) 40 µg/ml doxycycline for 3 hours. The expression of GFPmut2 or/and mCherry in strains WCL101, WCL103, and WCL105 was used as a control. (**B**) *Ca*Cdc11-GFPmut2 and mCherry-*Ca*Cdc10 were localized at the septum of strains WCL115 and WCL116, respectively. Co-localisation of mCherry-*Ca*Cdc10 and *Ca*Cdc11-GFPmut2 was visualized at the septum of the yeast form under a fluorescence microscope. White arrows indicate areas of fluorescent fusion at the septum. Blue arrows indicate the part of fluorescent fusion in vacuoles. The bar indicates 10 μm.

The septin family of proteins in *C. albicans*, including *Ca*Cdc3, *Ca*Cdc10, *Ca*Cdc11, *Ca*Cdc12, *Ca*Sep7, *Ca*Spr3, and *Ca*Spr28, are highly conserved cytoskeletal proteins with functions in cytokinesis^28^. The ring structure formed by septation during budding and filamentous growth is composed of four septin proteins, *Ca*Cdc3, *Ca*Cdc10, *Ca*Cdc11, and *Ca*Cdc12, and directs proper bud formation^29^. To use the fluorescent protein as a signal to label the ring during budding in *C. albicans*, the septin proteins *Ca*Cdc10 and *Ca*Cdc11 were incorporated into *Ca*Cdc11-GFPmut2 and mCherry-*Ca*Cdc10 fusion proteins whose expressions were regulated under the pWTN2 and pNIM1-NC Tet-on systems (Fig. S5), respectively. Cells in the WCL114 and WCL115 strains were capable of expressing the mCherry-*Ca*Cdc10 and *Ca*Cdc11-GFPmut2 fusion proteins, respectively. These cells were grown in YPD containing 40 µg/ml doxycycline, and the presence of mCherry-*Ca*Cdc10 and *Ca*Cdc11-GFPmut2 in the WCL114 and WCL115 strains was examined by western blotting (Fig. 3A). The induced expression and location of *Ca*Cdc11-GFPmut2 and mCherry-*Ca*Cdc10 in the cells after induction were assessed in parallel by the presence of a fluorescent signal at the septin ring by fluorescence microscopy. Focal fluorescence from both the GFPmut2 fusion and mCherry fusion proteins was observed, even though fluorescence was observed in the cytoplasm, particularly in cells from the WCL114 strain, due to the expression of rtTA driven by the strong *CaTDH3* promoter (Fig. 3B). To further test the co-localization of *Ca*Cdc11-GFPmut2 and mCherry-*Ca*Cdc10 at the septin ring in a cell, two Tet-on systems, pWTN2-mCherry-CDC10 and pNIM1-CDC11-GFPmut2, were incorporated and integrated at the *CaTDH3* and *CaADH1* loci to create the WCL116 strain. The induced expression of both the *Ca*Cdc11-GFPmut2 and mCherry-*Ca*Cdc10 fusion proteins in the presence of doxycycline was verified by western blotting (Fig. 3A). The colocalisation of GFPmut2 and mCherry at the septin ring detected by fluorescent microscopy (Fig. 3B) suggested that neither GFPmut2 nor mCherry influences the targeting of *Ca*Cdc11 and *Ca*Cdc10 to the septin ring. However, the high level of GFPmut2 and mCherry expression under the two Tet-on systems resulted in cytoplasmic diffusion of the cells (Fig. 3B, blue arrows). There are two possible explanations for this observation. One is the maintenance of homeostasis of the dynamic proteome through degradation processes, such as the ubiquitin-proteasome pathway^30^. After the degradation of *Ca*Cdc11-GFPmut2 and mCherry-*Ca*Cdc10, residual GFPmut2 and mCherry remained in the cytoplasm. As shown in Fig. 3A, some smearing in the samples from the WCL114 and WCL116 strains was observed, suggesting that degradation occurred. The other explanation for the observed cytoplasmic diffusion is that overexpressed *Ca*Cdc11-GFPmut2 and mChery-*Ca*Cdc10 accumulated in or co-localised at vacuoles (Fig. 3B, blue arrows). Because septins participate in the biogenesis of autophagosomes in yeast^31^, the overexpression of *Ca*Cdc10 and *Ca*Cdc11 could have prevented their correct localization. These results helped us to understand that gene overexpression under the Tet-on system not only exerts targeting effects but also brings about side effects due to the high dose of doxycycline (40 μg/ml).

### Establishment of a tetracycline-regulated split-mCherry for the BiFC assay in *C. albicans*

As the CUG codon is noncanonical in *C. albicans*, the CUG codons in exogenous reporter genes, such as fluorescent proteins and luciferase, must be optimized for their protein expression^32,33^. With regards to the use of a fluorescent protein, a red fluorescent protein variant, mCherry, was developed for use in the budding yeasts *Saccharomyces cerevisiae* and *C. albicans*, both of which surprisingly generate pink colonies (Fig. S3)^34^. The mCherry protein, a yeast-enhanced monomeric red fluorescent protein (yEmRFP), is an assembly of split amino acid fragments containing amino acids 1–159 (RN, the N-terminal fragment of mCherry) and 160–237 (RC, the C-terminal fragment of mCherry)^22^, which is similar to the split fragments of EGFP (amino acid sequence 1–157/158–238). Based on its protein complementation, monomeric structure and the formation of pink colonies, we used mCherry as a candidate fluorescence protein for the BiFC assay in *C. albicans*. To transfer the split fragments of mCherry into the pNIM1-NC and pWTN2 plasmids, intermediate cassettes consisting of the GFPmut2 coding sequence linked to the coding sequence of amino acids 160–237 of mCherry via the DNA encoding a triple GGGGS linker and the coding sequence of amino acids 1–159 of mCherry connected to the GFPmut2 coding sequence through the DNA encoding a triple GGGGS linker were cloned into pNIM1-NC and pWTN2 to generate pNIM1-RC-MCS and pWTN1-6H-MCS-RN, respectively. (see the Materials and Methods).

**Figure 4:**
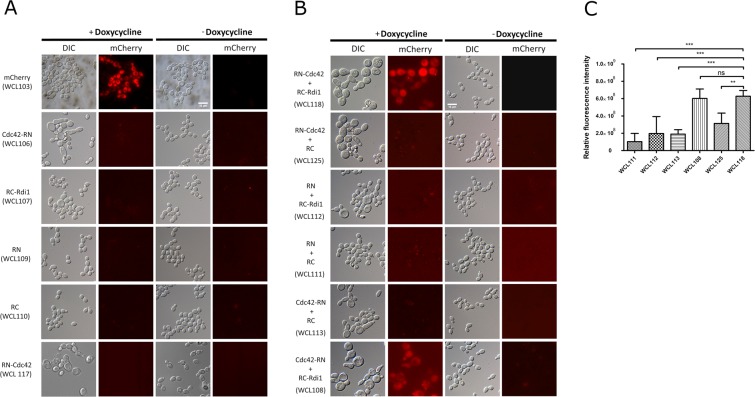
The mCherry-based BiFC system regulated by the Tet-on system in *C. albicans*. (**A**) Validation of split mCherry fragments unable to rejoin into a fluorescent complex. *C. albicans* cells from strains WCL103, WCL106, WCL107, WCL109, WCL110, and WCL117 were grown in YPD with and without 40 μg/ml doxycycline for 6 hours. The cells from strains WCL106, WCL107, and WCL117 increased in size. Red fluorescence in both the presence and absence of doxycycline was undetectable under fluorescent microscopy. The bar indicates 10 μm. (**B**) Examination of the mCherry-based BiFC system by the interaction of *Ca*Cdc42-*Ca*Rdi1. *C. albicans* cells from strains WCL111, WCL112, WCL113, WCL125, WCL108, and WCL118 were grown in YPD with and without 40 μg/ml doxycycline for 6 hours. Red fluorescence in the large cells of strains WCL118 and WCL108 was visualized by fluorescence microscopy. The other strain exhibited a background or undetectable signal. The bar indicates 10 μm. (**C**) Quantification of relative fluorescence intensity of the strains WCL111, WCL112, WCL113, WCL108, WCL125, and WCL118 with ImageJ. The values of bar charts derived from the integrated density of the original figures (Fig. S8) subtracting the background were analysed with one-way ANOVA with Bonferroni’s comparison test. ns: not statistically significant.

The highly conserved proteins Cdc42 and Rdi1 are known to interact to regulate polarized growth in eukaryotic cells^8,23,35,36^. These two partners have been applied to the BiFC assay in *S. cerevisiae*, and a structure of these proteins in complex was resolved via biophysical methods^8^. By analogy, Cdc42 and Rdi1 were incorporated into the Tet-on-based BiFC system. The coding sequence of *CaCDC42* was linked to the sequence encoding the N-terminal amino acids 1–159 of mCherry with a flexible linker containing the triple GGGGS peptide sequence, which was based on the pWTN1-6H-CDC42-RN plasmid (Fig. S6), while the coding sequence of *CaRDI1* was connected to a sequence encoding the C-terminal amino acids 160–237 of mCherry, which was based on the pNIM1-RC-RDI1 plasmid (Fig. S6). The C-terminus of Cdc42 interacts with Rdi1^37^. To avoid the interference of interaction between Cdc42 and Rdi1 when the C-terminus of Cdc42 fused with the N-terminus of mCherry derived from the pWTN1-6H-CDC42-RN plasmid was used, we constructed an alternate pWTN1-RN-CDC42 construct (Fig. S6), in which the sequence encoding the N-terminal amino acids 1–159 of mCherry was linked to the coding sequence of *CaCDC42* with a flexible linker containing the triple GGGGS peptide sequence. To examine doxycycline-induced protein expression with the Tet-on system by BiFC, strains (Table 1) capable of expressing each single component (WCL106, WCL107, WCL109, WCL110, and WCL117) and two components (WCL108, WCL111, WCL112, WCL113, WCL118, and WCL125) of the Tet-on system were grown in YPD containing 40 µg/ml doxycycline for 3 hours, and protein expression was verified by western blotting (Fig. S7). To detect the red fluorescence and eliminate possible background from the non-fluorescent fragment, strains capable of expressing a single component involved in BiFC (WCL106, WCL107, WCL109, WCL110, and WCL117) were grown in the presence or absence of doxycycline for 6 hours and analysed by fluorescence microscopy (Fig. 4A). As expected, red fluorescence was undetected with and without doxycycline induction in the WCL106, WCL107, WCL109, WCL110 and WCL117 strains, but red fluorescence displayed in the WCL103 capable of expressing mCherry in the presence of doxycycline (Fig. 4A).

Next, to test the emission of red fluorescence from the BiFC system, strains capable of expressing two components of the BiFC system (WCL108, WCL111,WCL112, WCL113, WCL118, and WCL125) were grown in YPD with or without doxycycline for 6 hours and examined under a fluorescence microscope. Only the cells of strains WCL108 and WCL118 (Cdc42-RN + RC-Rdi1 and RN-Cdc42 + RC-Rdi1, respectively) displayed red fluorescence following growth under doxycycline-induced conditions (Fig. 4B). However, the other strains, WCL125, WCL113, WCL112, and WCL111, exhibited very weak fluorescent signals following growth in the presence of doxycycline (Fig. 4B). The weak fluorescent signal was thought to be background fluorescence because the overexpressed split fragments of mCherry had accumulated in the cells. To validate the formation of fluorescence dependent on the interaction of *Ca*Cdc42 and *Ca*Rdi1, randomly selected images of 5 fields from the slides on which the strains capable of expressing split-fragments of BiFC under fluorescence microscope were captured (Fig. S8) and quantified the relative fluorescence intensity. By using statistical analysis (Table S3), the relative fluorescence intensity of the strains WCL108 and WCL118 showed a significant difference from the others (Fig. 4C). The unexpected fluorescence occurred in the degraded cells of the strains, likely being the result of the aggregation of the released proteins from the dead cells. Reconstitution of functional mCherry indicated the interaction of the *Ca*Cdc42 and *Ca*Rdi1 fusion proteins. The reconstituted fluorescence via *Ca*Cdc42-*Ca*Rdi1 interaction in the WCL118 strain in the presence of doxycycline detected by BiFC was dispersed in the cells. Although Cdc42 and Rdi1 were shown to co-localize in the cytoplasm, at the tips of small buds, and at the mother-bud neck region in the budding yeast^38^ as an unicellular model, the focal point of the *Ca*Cdc42-*Ca*Rdi1 interaction in our study was not clear without the use of confocal microscopy. Interestingly, the overexpression of *Ca*Cdc42 (WCL113 and WCL125), *Ca*Rdi1(WCL112), or both fusion proteins (WCL108 and WCL118) enlarged the cells grown under doxycycline-induced conditions. The phenotypes of these cells resembled those of budding yeast cells overexpressing Cdc42 or Rdi1, in which the cells were increased in size due to the loss of cell polarity^39,40^. Reconstitution of the split fragments of mCherry in strains WCL118 and WCL108 still occurred in the presence of doxycycline, even if the overexpression of the fusion proteins altered the phenotype. Based on these observations, particularly the results of fluorescence microscopy, we concluded that our newly established BiFC system functions in *C. albicans* to assay protein-protein interactions.

## Discussion

In this study, we took advantage of the dominant selectable marker *CaHygB*, which comprises the *C. albicans* codon-optimized coding sequence of hygromycin B phosphotransferase from *E. coli* driven by the *C. albicans TEF2* (*CaTEF2*) promoter^21^, and a tetracycline-regulated gene expression system in *C. albicans*^16^ to create a new version of the cassette in the form of two plasmids, pWTN1 and pWTN2 (Fig. S1). Each of these plasmids is capable of expressing GFPmut2 or mCherry as a tool for molecular manipulation in *C. albicans*. Together with the pNIM1-NC plasmid (Fig. S1), which was modified from the pNIM1 plasmid^16^ carrying the *CaSAT1* selection marker for nourseothricin resistance and is capable of expressing GFPmut2, the use of the pWTN2 plasmid (or their derivatives) to generate *C. albicans* strains confirms that this system, which has the benefit of dominant selection markers, can be used to study the regulation of two proteins simultaneously in cells with diverse morphologies (Fig. 1). Regardless of the identity of the gene regulated by one or two Tet-on systems, GFPmut2 and mCherry were expressed in a dose-dependent manner (Fig. 2). This new system appears to be superior to the current expression system^15^ for the concurrent dominantly selection and inducible expression of two proteins. A test of the system with the *Ca*Cdc10 and *Ca*Cdc11 components from the septin family via the production of mCherry-*Ca*Cdc10 and *Ca*Cdc11-GFPmut2 fusion proteins under tetracycline-regulated promoters showed the co-localization of GFPmut2 and mCherry at the septin ring (Fig. 3). These results indicate the feasibility of introducing the new system to study the function of proteins when localization and imaging are conducted. Furthermore, to extend the new system for the assessment of protein-protein interactions, the BiFC assay was established. A derivative of the DsRed fluorescent protein, mCherry, was divided into two fragments, with one fragment containing amino acids 1–159 and another containing amino acids 160–237, which were generated as two fusion proteins in the Tet-on system^22^. The well-established interacting proteins *Ca*Cdc42 and *Ca*Rdi1, which function in polarized growth^23^, were used to validate the feasibility of BiFC because their homologs in *S. cerevisiae* have been shown to interact with the BiFC assay^8^. Indeed, red fluorescence was observed in the WCL108 and WCL118 strains in the presence of doxycycline due to the interaction between *Ca*Cdc42 and *Ca*Rdi1.

The newly developed Tet-on system functions in a characteristic dose-dependent manner (Fig. 2) and responds immediately to the presence of doxycycline for at most one hour. The regulation of one Tet-on system, with a reverse tetracycline-regulated transactivator (rtTA) and a set of tetracycline response elements, is simple. Because of the constitutive expression of rtTA via two different promoters of *CaADH1* and *CaTDH3* in these two Tet-on systems, the level of doxycycline-inducible products differs, like the level of GFPmut2 in the WCL101 and WCL102 strains (Fig. 2). The variable strength of the promoters was validated in *S. cerevisiae*, in which the *TDH3* promoter was proven to be stronger than the *ADH1* promoter^41^. This result is consistent with ours, in which the level of GFPmut2 expression was different depending on the promoter (Fig. 2). When rtTA or the dose of doxycycline increases, or the copy number of the target gene is higher, the target protein is expressed at higher levels. Extremely high levels of protein expression result in protein accumulation in the cell, which affects homeostasis^42^, even though some of the proteins may still retain their normal function in the cell. In the case of mCherry-*Ca*Cdc10 and *Ca*Cdc11-GFPmut2, mCherry and GFPmut2 were found to colocalise in the cytoplasm and at the septin ring, but their colocalisation was somewhat ambiguous due to the build-up of exceedingly higher amounts of protein in all parts of the cell, which masked focal fluorescence at the septin ring (as in WCL116 in Fig. 3). However, focal fluorescence was easily observed at the septin ring from cells of the WCL114 and WCL115 strains expressing a single fluorescent fusion protein controlled by one Tet-on system, suggesting that induction conditions may need to be optimized in terms of both time and dose to overcome possible functional disturbances of the fusion protein.

**Figure 5:**
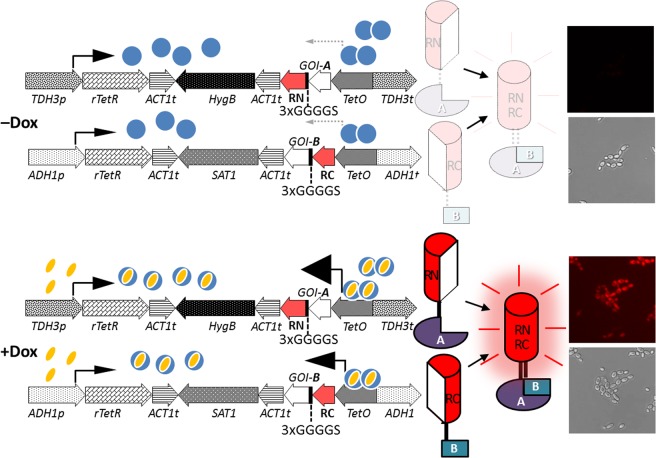
Establishment of tetracycline-induced bimolecular fluorescence complementation to detect protein-protein interaction in *Candida albicans*. Doxycycline (Dox), a tetracycline analogue, is used to induce gene expression. The sizes of the arrowheads indicate the relative strength of transcriptional activation.

In a previous study, *S. cerevisiae* and *C. albicans* containing mCherry were developed, and, surprisingly, pink colonies of the derivative strains were observed^34^. Consequently, we thought that this fluorescent protein could be used in the BiFC assay and applied to detect protein-protein interactions without any fluorescence equipment but with the visible spectrum. However, although the colonies of the WCL108 and WCL118 strains were not pink, red fluorescence was observed. We believe that the structure of the assembled complex is somewhat different from that of the native protein. We used *Ca*Cdc42 and *Ca*Rdi1 in the BiFC assay in *C. albicans* as the interruption of *Ca*Cdc42 by a cell-penetrating peptide has been shown to inhibit the yeast-to-filament transition^43^, which can potentially be used as a drug target. Recently, a BiFC system^44^ based on a set of *C. albicans*-optimized plasmids containing amino acid biosynthetic genes as selectable markers that uses the *C. albicans MET3* promoter to drive expression of split fusion fluorescence proteins was developed. However, this strategy relies on auxotrophic strains for selection, and repression of the repressible *MET3* promoter requires methionine and cysteine, which may interfere with or be interfered by the metabolism of the growing cells. Compared with the system mentioned above, our system, in which dominant selection markers and a Tet-on system are used to drive the expression of fusion proteins containing split fragments of fluorescence proteins, is preferential, as shown in a schematic representation (Fig. 5). This newly developed BiFC system can be used not only to assess function but also to conduct genome-wide screening for protein interacting partners in *C. albicans*. Additionally, once the interaction between two proteins is verified by the BiFC assay, it can be applied further to screen small molecule, peptide, and protein drugs to block their interaction for therapeutic purposes.

## Supplementary information

Supplementary information.
