# Supplementary material for: Establishment of tetracycline-regulated bimolecular fluorescence complementation assay to detect protein-protein interactions in Candida albicans
Source: Sci Rep. 2020 Feb 19;10:2936. doi: 10.1038/s41598-020-59891-7 (PMC7031294; doi:10.1038/s41598-020-59891-7)
Supplement: Supplementary file 1 — Supplementary information. [file 41598_2020_59891_MOESM1_ESM.pdf]

Supplementary Information

Establishment of tetracycline-regulated bimolecular fluorescence complementation assay to detect protein-protein interactions in *Candida albicans*

Running title: BiFC in *Candida albicans*

Wei-Chung Lai[1§], H. Sunny Sun[2], and Jia-Ching Shieh[1, 3, 4]*

[1]Department of Biomedical Sciences, Chung Shan Medical University, Taiwan, R.O.C.

[2]Institute of Molecular Medicine, College of Medicine, National Cheng Kung University, Taiwan, R.O.C.

[3]Department of Medical Research, Chung Shan Medical University Hospital, Taiwan, R.O.C.

[4]Immunology Research Centre, Chung Shan Medical University

*Corresponding author:

Dr. Jia-Ching Shieh

Department of Biomedical Sciences, Chung Shan Medical University, No. 110, Sec.1, Jianguo N. Road, Taichung City 40201, Taiwan, R.O.C.

Phone: +886-4-24730022, ext. 11806
Fax: +886-4-2475-7412
E-mail: jcs@csmu.edu.tw

[§]Current address:

Dr. Wei-Chung Lai

Institute of Molecular Medicine, National Cheng Kung University Medical College, Taiwan, R.O.C.

E-mail: workman_pum250@hotmail.com

Supplementary information

List of Contents

1. Supplemental Materials and Methods
2. Supplemental Figure legends
3. Supplemental Figure 1 (Figure S1)
4. Supplemental Figure 2 (Figure S2)
5. Supplemental Figure 3 (Figure S3)
6. Supplemental Figure 1 (Figure S4)
7. Supplemental Figure 1 (Figure S5)
8. Supplemental Figure 1 (Figure S6)
9. Supplemental Figure 1 (Figure S7)
10. Supplemental Figure 1 (Figure S8)
11. Supplemental Table 1 (Table S1)
12. Supplemental Table 2 (Table S2)
13. Supplemental Table 2 (Table S3)

1. Supplemental Materials and Methods

Construction of Tet-on Plasmids

We aimed to set up a Tet-on-based BiFC assay; thus, we developed an additional Tet-on system with hygromycin B resistance in *C. albicans* following the construction of pNIM1 [1]. To efficiently utilize the pNIM1 vector described in our previous study [2], a pair of primers, CaGFP-N-MCS and CaGFP-C-MCS, was designed to incorporate multiple cloning sites to the 5’ and 3’ ends of the GFPmut2-coding sequence by PCR, followed by digesting with *Sal*I/*Bcl*I and cloning back into *Sal*I/*Bgl*II-linearized pNIM1 to obtain pNIM1-NC (Figure S1). To construct the additional pWTN2 plasmid for red fluorescent validation, the assembly was divided into three steps. **(1)** Construction of pBSII-TDH3p-rtTA-ACT1t. The pWTN2 plasmid was designed for gene targeting at the *CaTDH3* locus due to its promoter, which is capable of constitutively driving reverse tetracycline-controlled transactivator (rtTA) expression. This promoter was amplified with the TDH3 SacII F and TDH3 XbaI R primers from genomic wild-type DNA and subsequently cloned into pBluescriptII SK(+) (pBSII) digested with *Sac*II/*Xba*I to create pBSII-CaTDH3p. The rtTA coding sequence was coupled to the *CaACT1* transcription terminator released from pNIM1, subcloned into pBSII-TDH3p and then digested with *Xba*I/*Pst*I to produce pBSII-TDH3p-rtTA-ACT1t. **(2)** Construction of pBSII-SFS-HygB. To create a backbone vector with hygromycin B resistance for the assembly of the components in the tetracycline-regulated system, the pSFS2A plasmid [3] was digested with *Sal*I to release a

part of the DNA fragment containing the *Spe*I site within the CaSAT1 gene and ligated into itself to obtain pSFS2A-SpeI with one remaining SpeI site. To replace the backbone of pBSII, the DNA cassette of pSFS2A-SpeI between two FRT sequences was digested with *Kpn*I/*Sac*I and subcloned into pBSII to generate pBSII-SFS2A-SpeI. To incorporate more suitable restriction enzyme sites flanking the FRT sequences, two DNA duplexes formed by two pairs of primers, MCS3 F/MCS3 R and MCS4 F/MCS4 R, were sequentially cloned into pBSII-SFS2A-SpeI via *Spe*I/*Sac*I and then via *Kpn*I/*Xho*I digestion to become pBSII-SFS-MCS4. The synthetic hygromycin B gene (*CaHygB*) was amplified with the HygB BglII F and HygB NsiI R primers using pYM70 [4] as a DNA template, cut out with *Bgl*II/*Nsi*I, and cloned into pBSII-SFS-MCS4 digested with *Bam*HI/*Pst*I to release a part of the SAT1-flipper to generate pBSII-SFS-HygB. **(3)** Construction of pCR2-TDH3t-TetO7-miniOP4-GFPt. To construct seven copies of the tetracycline operator (*tet*O), a unit of the *tet*O adaptor formed by annealing with a pair of primers, TetO F and TetO R, was cloned into pBSII-HA-2xStrepII (our unpublished data) digested with *Pme*I/*Sac*I to produce pBSII-TetO1. Then, another adaptor was digested with *Pme*I/*Sal*I and cloned into pBSII-TetO1 digested with *Pme*I/*Xho*I to generate pBSII-TetO2. This step was repeated five times to construct pBSII-TetO7. We used the multiple cloning site (MCS) of pCR2.0 for plasmid building by digesting TetO7 with *Kpn*I/*Sac*I and subcloning into pCR2.0 to generate pCR2-TetO7. To fuse the minimal *CaOP4* promoter (miniOP4) to TetO7, the promoter was

PCR-amplified with the miniOP4 SpeI F and miniOP4 XhoI R primers using pNIM1 as a DNA template; the construct was then digested with *Xho*I/*Spe*I and cloned into pCR2-TetO7 digested with *Sal*I/*Spe*I to become pCR2-TetO7-miniOP4. A DNA fragment homologous to *CaTDH3* for gene targeting was amplified with the TDH3t PmeI F2 and TDH3t KpnI R2 primers, digested with *Pme*I/*Kpn*I, and cloned into pCR2-TetO7-miniOP4 to generate pCR2-TDH3t-TetO7-miniOP4. Similar to pNIM1, a GFPmut2-coding sequence linked to the *CaACT1* transcriptional terminator was amplified with the GFPt XbaI F and GFPt SpeI R primers from the pNIM1-NC template, digested with *Xba*I/*Spe*I, and cloned into pCR2-TDH3t-TetO7-miniOP4 to produce pCR2-TDH3t-TetO7-miniOP4-GFPt. To assemble the pWTN1 plasmid, a prototype of pWTN2, from these three constructs, the DNA fragment comprising TDH3p-rtTA-ACT1t was released from the pBSII-TDH3p-rtTA-ACT1t plasmid via *Pst*I/SacII digestion and was cloned into pBSII-SFS-HygB linearized with *Pst*I/*Sac*II to create pBSII-TDH3p-rtTA-HygB; then, another DNA fragment containing TDH3t-TetO7-miniOP4-GFPt was released from pCR2-TDH3t-TetO7-miniOP4-GFPt via *Kpn*I/*Age*I digestion and cloned into pBSII-TDH3p-rtTA-HygB to generate pWTN1 (Figure S1). To replace the GFPmut2-coding sequence of pWTN1with yEmRFP (yeast enhanced red fluorescent protein, as well as mCherry) [5], which is a codon-optimized RFP for *C. albicans*, a pair of primers, mRFP NotI F and mRFP AatII R and pFA-yEmRFP-CmLEU2-2μ (our unpublished data), was used as a DNA template to amplify a coding sequence of yEmRFP; the

construct was digested with *Not*I/*Aat*II and cloned into pWTN1 to generate pWTN2 (Figure S1). For fluorescent labelling of septin, the coding sequences of *Ca*Cdc10 and *Ca*Cdc11 were digested with *Nhe*I/*Xho*I and *Aat*II/*Bgl*II from pTET25M-CDC11-GFP and pTET25M-RFP-CDC10 (our unpublished data) and cloned into pNIM1-NC and pWTN2, respectively, to generate pNIM1-CDC11-GFPmut2 and pWTN2-mCherry-CDC10 (Figure S2).

Design of the BiFC assay

To fit the Tet-on systems for the BiFC assay, mCherry was used as a reporter in this study. Split-mCherry, which is capable of reassembling, was divided into an N-terminal fragment containing amino acids 1-159 and a C-terminal fragment containing amino acids 160-237 [6]. The triple repeats of the GGGGS amino acid sequence were used to increase structural flexibility between a target gene and fragments of the reporter. To create DNA segments encoding the GGGGS amino acid sequence, we designed a pair of primers, GS F and GS R, to form a single unit of GS adaptor by annealing to each other and subsequently ligating into pBSII digested with *Eco*RV/*Bam*HI to become pBSII-GS1. Then, two additional GS adaptors were sequentially cloned into pBSII-GS1 digested with *Eco*RV/*Bgl*II to obtain pBSII-GS3, which was used as an intermediate vector for fusion with the targets and split fragments in the Tet-on system. To construct pWTN1-6H-MCS-RN, which is capable of expressing the

C-terminal end of the target protein with a polyhistidine tag (6H) fused to the N-terminal fragment of mCherry (RN), both the DNA sequences encoding 6H and RN were incorporated into pWTN1. The DNA adaptor encoding six histidine residues made by a pair of primers, WTN1-His F and WTN1-His R, was cloned into pWTN1 digested with *Spe*I/*Eco*RV to obtain pWTN1-6H. Subsequently, pWTN1-6H was digested with *Kpn*I/*Bgl*II and ligated into *Kpn*I/*Bgl*II-digested pBSII-GS3 to become pBSII-WTN1-6H-GS3. The DNA fragment encoding the N-terminal fragment of mCherry was PCR-amplified with primers, mRFP N BamHI F and mRFP N NotI R, and with pWTN2 as the DNA template and HiFi DNA polymerase (Kapa Biosystems), the fragment was then cloned into *Bam*HI/*Not*I-digested pBSII to obtain pBSII-RN. Next, the WTN1-6H-GS3 DNA fragment was released from pBSII-WTN1-GS3 by digesting with *Kpn*I/*Bam*HI and was cloned into pBSII-RN to create pBSII-WTN1-6H-GS3-RN. Finally, the WTN1-6H-GS3-RN DNA fragment was released from this plasmid digested with *Kpn*I/*Stu*I and was cloned into pWTN1 to generate pWTN1-6H-MCS-RN. To create a vector capable of expressing the N-terminal fragment of mCherry to use as a control, this encoding fragment was amplified with a pair of primers, RedN KpnI F and mRFP N NotI R, following *Spe*I/*Stu*I digestion and cloned into pWTN1 to generate pWTN1-RN. To construct another vector, pNIM1-RC-MCS, which is capable of producing the N-terminal end of the target protein fused to the C-terminal fragment of mCherry (RC), the pNIM1-NC was used and modified by a similar strategy described above.

The DNA fragment released from pNIM1 by *Kpn*I/*Sal*I digestion was cloned into *Kpn*I/*Sal*I-digested pBSII-GS3 to obtain pBSII-NIM1-GS3. The DNA encoding the C-terminal fragment of mCherry was PCR-amplified with the primers using pWTN2 as a DNA template; after *Xho*I/*Bam*HI digestion, the fragment was cloned into *Sal*I/*Bgl*II-digested pBSII-NIM1-GS3 to produce pBSII-NIM1-RC-GS3. The NIM1-RC-GS3 DNA fragment was released from this plasmid by *Kpn*I/*Spe*I digestion and cloned into *Kpn*I/*Spe*I-digested pCR2-TDH3t-OP-GFPt to produce pCR2-NIM1-RC-GS3-GFPt. Finally, the NIM1-RC-GS3-GFPt DNA fragment released from pCR2-NIM1-RC-GS3-GFPt digested with *Kpn*I/*Stu*I was cloned into *Kpn*I/*Stu*I-digested pNIM1-NC to generate pNIM1-RC-MCS. To generate a vector capable of expressing the C-terminal fragment of mCherry as a control, this encoding fragment was amplified and produced with a pair of primers, mRFP C XhoI F and mRFP C BamHI R, and with pWTN2 as a template; after *Xho*I/*Bam*HI digestion, the fragment was cloned into *Sal*I/*Bgl*II-digested pNIM1 to become pNIM1-RC. Then, the coding sequences of *CaCDC42* and *CaRDI1* were amplified with the CDC42 EcoRV F and CDC42 BglII R, and RDI1 SpeI F and RDI1 StuI R primers, respectively, and cloned into pWTN1-6H-MCS-RN and pNIM1-RC-MCS to produce pWTN1-6H-CDC42-RN and pNIM1-RC-RDI1 (Figure S3). To construct a vector, pWTN1-RN-CDC42 (Figure S3), capable of expressing the N-terminal mCherry fusion to CDC42, a PCR-amplified DNA fragment encoding amino acids 1- 159 of the mCherry protein by a pair of primers, RedN

KpnI F and RedN EcoRV R, was cloned into the pBSII-GS3 plasmid linearized with *Kpn*I and *Eco*RV to obtain pBSII-RN-GS3. Next, a PCR-amplified coding fragment of CDC42 by a pair of primers, CaCDC42 BamHI F and CaCDC42 NotI R, was cloned into pBSII-RN-GS3 linearized with *Bam*HI and *Not*I to obtain pBSII-RN-GS3-CDC42. Finally, the DNA fragment containing the RN-GS3-CDC42 region was released by *Spe*I/*Stu*I and cloned into pWTN2 digested with *Spe*I/*Stu*I to obtain pWTN1-RN-CDC42 (Figure S3).

References

1. Park, Y. N. & Morschhauser, J. Tetracycline-inducible gene expression and gene deletion in Candida albicans. *Eukaryot Cell* **4**, 1328-1342, doi:10.1128/EC.4.8.1328-1342.2005 (2005).
2. Lai, W. C. *et al.* Construction of Candida albicans Tet-on tagging vectors with a Ura-blaster cassette. *Yeast* **28**, 253-263, doi:10.1002/yea.1833 (2011).
3. Reuss, O., Vik, A., Kolter, R. & Morschhauser, J. The SAT1 flipper, an optimized tool for gene disruption in Candida albicans. *Gene* **341**, 119-127, doi:10.1016/j.gene.2004.06.021 (2004).
4. Basso, L. R., Jr. *et al.* Transformation of Candida albicans with a synthetic hygromycin B resistance gene. *Yeast* **27**, 1039-1048, doi:10.1002/yea.1813 (2010).
5. Keppler-Ross, S., Noffz, C. & Dean, N. A new purple fluorescent color marker for genetic studies in Saccharomyces cerevisiae and Candida albicans. *Genetics* **179**, 705-710, doi:10.1534/genetics.108.087080 (2008).
6. Fan, J. Y. *et al.* Split mCherry as a new red bimolecular fluorescence complementation system for visualizing protein-protein interactions in living cells. *Biochem Biophys Res Commun* **367**, 47-53, doi:10.1016/j.bbrc.2007.12.101 (2008).

2. Supplementary figure legends

Figure S1. The maps of pNIM1-NC, pWTN1, and pWTN2 used in this study.

(A) The functional elements of the Tet-on system are shown to present the cassettes of pNIM1-NC, pWTN1 and pWTN2. *ADH1p*: *CaADH1* promoter, *rTetR*: the coding sequence of rtTA, *ACT1t*: *CaACT1* transcription terminator, *HygB*: *CaHygB* serves as a dominant selectable marker offering hygromycin B resistance, *TetO*: a Tet operator sequence fused to a minimal *CaOP4* promoter, *ADH1t*: a part of *CaADH1* for homologous recombination. Restriction enzyme sites, *Sac*II and *Kpn*I, were used to linearise the cassettes. **(B)** The multiple cloning sites (MCS) of pNIM1-NC are shown. **(C)** The MCS of pWTN1 are shown. **(D)** The MCS of pWTN2 are shown. TAA was designed as the stop codon of coding sequence.

Figure S2. Confirmation of the strains carrying the cassettes, pNIM1-NC, pWTN1, pWTN2, or both by yeast colony PCR. The 1082 bp of amplicons were expanded with primers, pNIM1-inte F and pNIM1-inte R, to indicate the *CaADH1* integration of pNIM1-NC in WCL101. The 1336 bp of amplicons were expanded with primers, CaTDF3 F and pNIM1-inte R, to indicate the *CaTDH3* integration of pWTN1 in WCL102 or pWTN2 in WCL103. Two amplicons were obtained simultaneously from WCL104 and WCL105 with

multiplex PCR with primers, pNIM1-inte F, pNIM1-inte R and CaTDH3 F. SC5314 was used as negative control.

Figure S3. Pink colonies of WCL103 and WCL 105 under doxycycline-induced conditions. The cells of the strains, WCL101, WCL102, WCL103, WCL104, WCL105 and SC5314 were grown onto synthetic minimal medium with and without 40 μg/ml doxycycline at 30℃ and 37℃ for 2 days. The colonies of WCL103 and WCL105 turned into pink, but the other strains sustained as white. The enlarged images "**a**" and "**b**" from the original images at 37℃ in the presence of doxycycline.

Figure S4. The triplicate western blots to quantify the expression levels of fluorescent proteins in the strains, WCL101, WCL102, WCL103, WCL104, andWCL105, with a variety of doxycycline doses. Dox: the dose of doxycycline. (A) The western blots used to analyse the GFPmut2 expression of the stains WCL101, WCL102, WCL104, and WCL105 with anti-GFP antibodies. (B) The western blots used to detect the mCherry expression of the strains WCL103 and WCL105 with anti-mCherry antibodies. As the strain WCL105 is capable of expressing GFPmut2 and mCherry in the presence of doxycycline, two western blots were used, as shown in (A) and (B), respectively. The red cropped blots were those used in Figure 2 as representatives. The fold changes of the expression of GFPmut2 and mCherry

from triplicate blots were listed in Table S1. Statistical analyses proceeded with one-way ANOVA with Bonferroni's comparison test listed in Table S2.

Figure S5. The maps of pNIM1-CDC11-GFP and pWTN2-mCherry-CDC10.

The coding sequence of *CaCDC11* was cloned into pNIM1-NC with *Xho*I and *Nhe*I, and the coding sequence of CaCDC10 was cloned into pWTN2 with *Aat*II and *Bgl*II.

Figure S6. The maps of pWTN1-6H-CDC42-RN, pWTN1-RN-CDC42, and pNIM1-RC-RDI1. RN: the coding sequence of N-terminus of mCheery, RC: the coding sequence of C-terminus of mCherry, 3xGGGGS: triple repeats of the coding sequence translated into amino acids GGGGS. The coding sequence of *CaCDC42* was cloned into pWTN1-6H-RN with *Eco*RV and *Bgl*II and into pWTN1-RN with *Spe*I and *Stu*I. The coding sequence of *CaRDI1* was cloned into pNIM-RC with *Spe*I and *Stu*I.

Figure S7. Verification of the strains expressing one or two components of the mCherry-based BiFC assay under the doxycycline-induced conditions by western blotting. (A) The cells of the strains, as indicated in the figure, were grown in YPD containing 40 µg/ml of

doxycycline for 3 hours before extracting the total lysates for 16% SDS-PAGE and western blotting with an anti-mCherry antibody. Each emerged band represents the indicated essential component in the BiFC assay (shown in brackets). The total lysate extracted from the wild-type SC5314 is used as a negative control. ß-actin is used as a loading control. (B) The cells of the indicated strains were grown in YPD containing 40 µg/ml of doxycycline for 3 hours. The total lysates were separated with 12% SDS-PAGE, followed by western blotting analysis with an anti-mCherry antibody. Arrows indicate the distinguishable *Ca*Cdc42-RN, RN-*Ca*Cdc42, and RC-*Ca*Rdi1 fusions on the blots. The Ponceau S staining shows the equal loading of the blot.

Figure S8. The fluorescent images from strains WCL111, WCL112, WCL113, WCL108, WCL125, and WCL118. The images of five fields from each strain were randomly selected. These images were used in the quantification of the relative fluorescence intensity. In the fields of the strains WCL111, WCL112, and WCL125, the degraded cells showed unexpected fluorescence (white arrows). Scale bar indicates 10 µm. The raw data and statistical analyses of one-way ANOVA with Bonferroni's comparison test were listed in Table S3.

Figure S1.

A

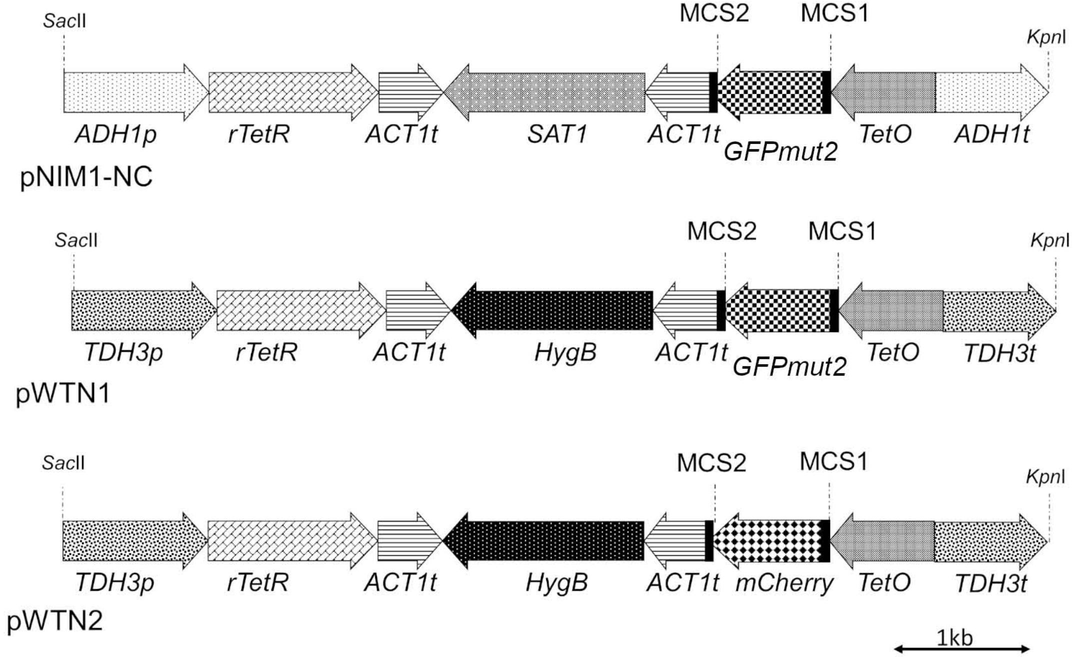

B

pNIM1-NC

MCS1

OP4 minimal promoter
TACGTTAACTCCTACACACATACAAATATAAATA

GFPmut2
GTC GAC TCG AGA TAT CCA GCT AGC GGC CGC GTG ACC ATG AGT AAG
*Sal*I *Xho*I *Eco*RV *Nhe*I *Not*I

MCS2

GFPmut2
GGC ATG GAT GAA CTA TAC AAA
STOP
GGT CCG GAC GTC AGC TTA AGA TCT AGG CCT GCA TAA TGA TCT
*Bsp*EI *Aat*II *Afl*II *Bgl*II *Stu*I

C

pWTN1

MCS1

OP4 minimal promoter
TACGTTAACTCCTACACACATACAAATATAAATA

GFPmut2
ACT AGT GTC GAC TCG AGA TAT CCA GCT AGC GGC CGC GTG ACC ATG AGT AAG
*Spe*I *Sal*I *Xho*I *Eco*RV *Nhe*I *Not*I

MCS2

GFPmut2
GGC ATG GAT GAA CTA TAC AAA
STOP
GGT CCG GAC GTC AGC TTA AGA TCT AGG CCT GCA TAA TGA TCT
*Bsp*EI *Aat*II *Afl*II *Bgl*II *Stu*I

D

pWTN2

MCS1

OP4 minimal promoter
TACGTTAACTCCTACACACATACAAATATAAATA

mCherry
ACT AGT GTC GAC TCG AGA TAT CCA GCT AGC GGC CGC GTG ACC ATG AGT AAG
*Spe*I *Sal*I *Xho*I *Eco*RV *Nhe*I *Not*I

MCS2

mCherry
GGC ATG GAT GAA CTA TAC AAA
STOP
GGT CCG GAC GTC AGC TTA AGA TCT AGG CCT GCA TAA TGA TCT
*Bsp*EI *Aat*II *Afl*II *Bgl*II *Stu*I

Figure S2

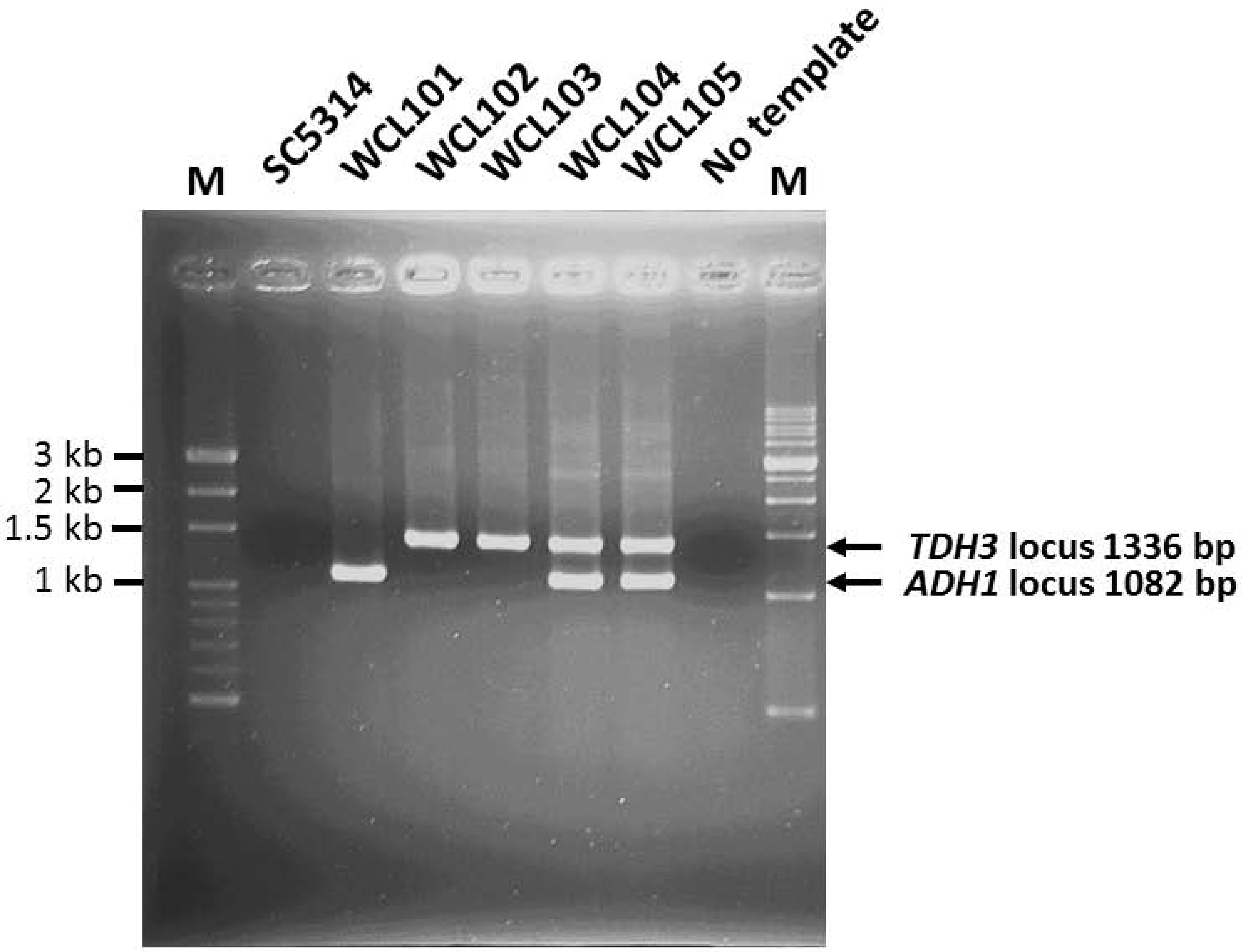
M
SC5314
WCL101
WCL102
WCL103
WCL104
WCL105
No template
M
3 kb
2 kb
1.5 kb
1 kb
TDH3 locus 1336 bp
ADH1 locus 1082 bp

Figure S3

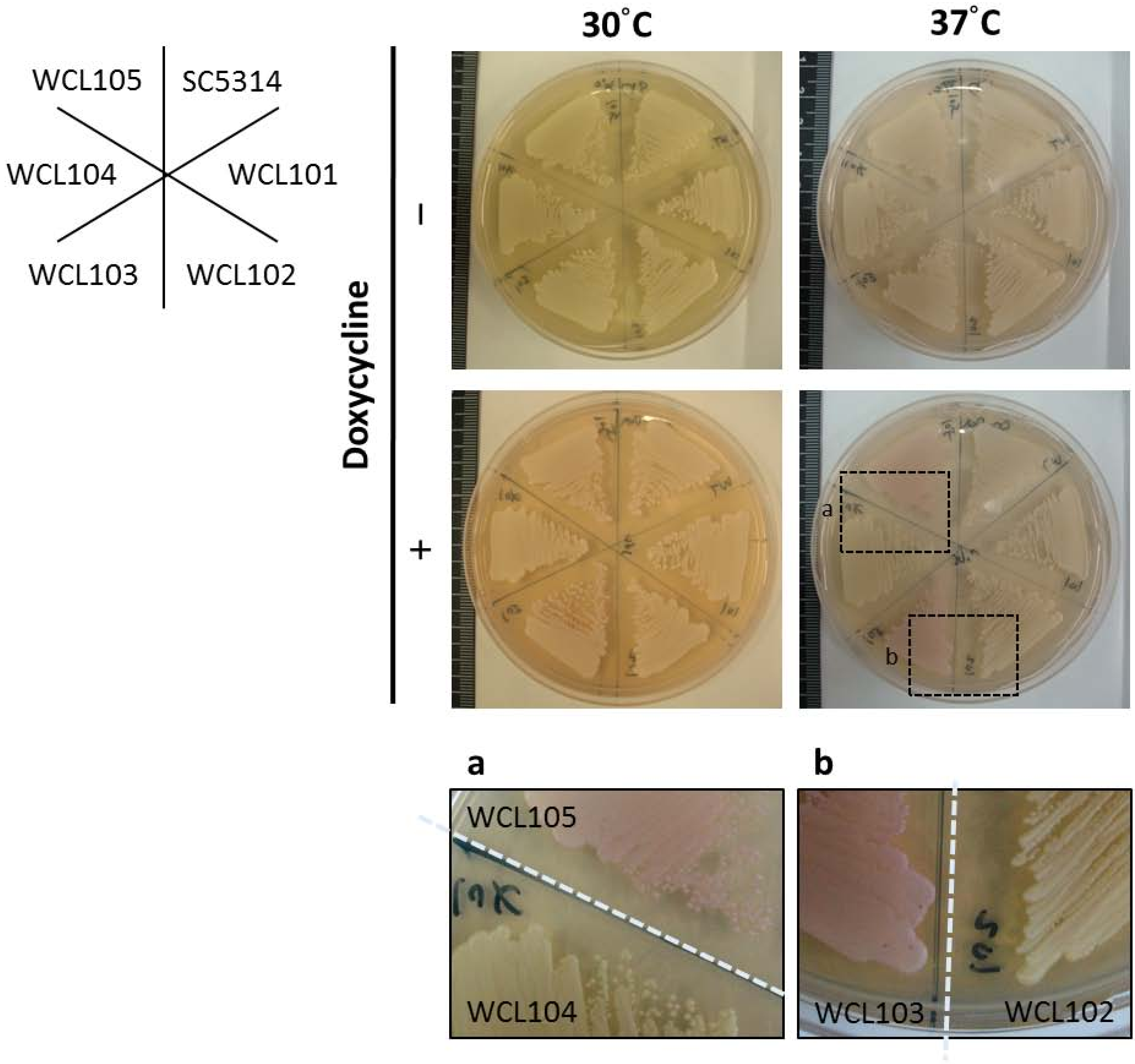
WCL105
SC5314
WCL104
WCL101
WCL103
WCL102
30°C
37°C
Doxycycline
–
+
a
b
a
WCL105
WCL104
b
WCL103
WCL102

Figure S4

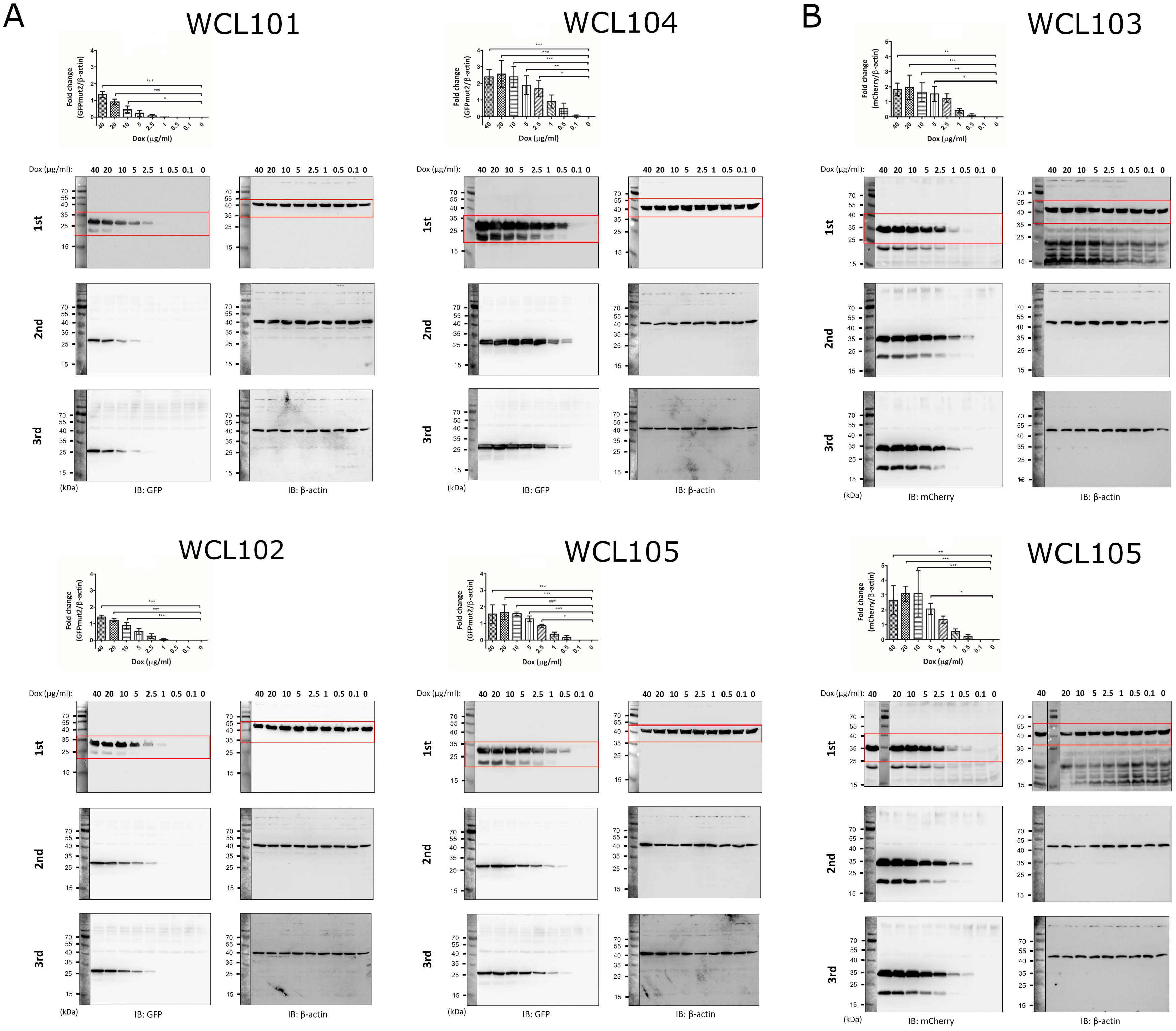

A
B
WCL101
WCL104
WCL103
WCL102
WCL105
WCL105
Fold change (GFPmut2/β-actin)
Fold change (mCherry/β-actin)
Dox (μg/ml)
40 20 10 5 2.5 1 0.5 0.1 0
1st
2nd
3rd
(kDa)
IB: GFP
IB: β-actin
IB: mCherry

Figure S5

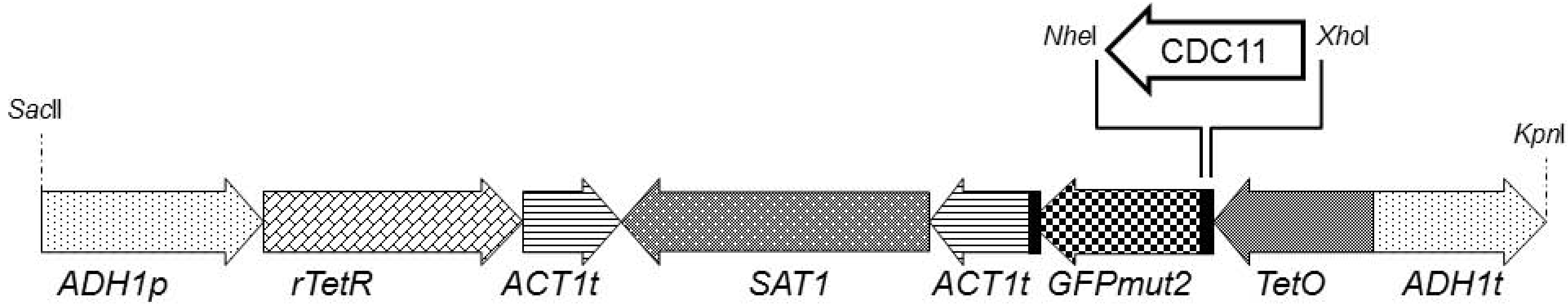

pNIM1-CDC11-GFP

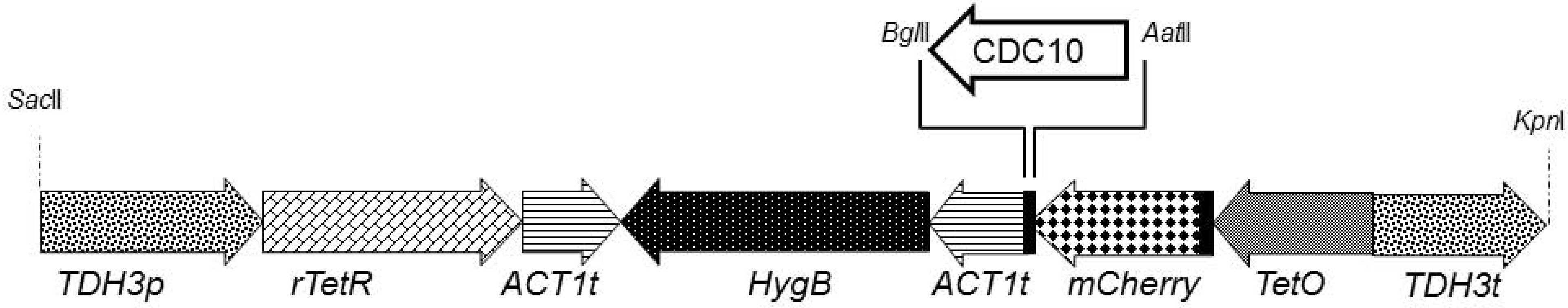

pWTN2-mCherry-CDC10

Figure S6

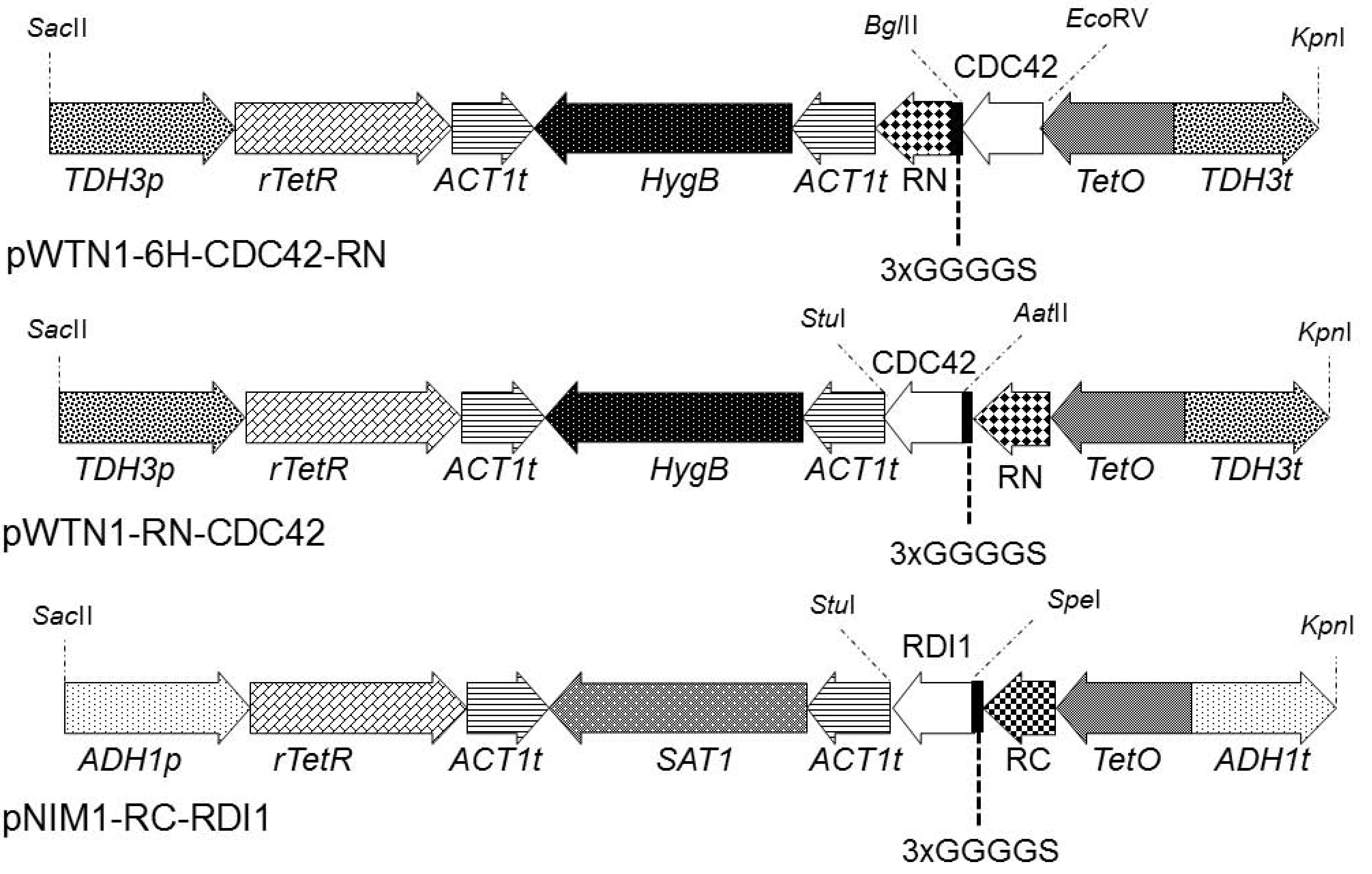
SacII
BglII
EcoRV
KpnI
CDC42
TDH3p
rTetR
ACT1t
HygB
ACT1t
RN
TetO
TDH3t
3xGGGGS
pWTN1-6H-CDC42-RN
SacII
StuI
AatII
KpnI
CDC42
TDH3p
rTetR
ACT1t
HygB
ACT1t
RN
TetO
TDH3t
3xGGGGS
pWTN1-RN-CDC42
SacII
StuI
SpeI
KpnI
RDI1
ADH1p
rTetR
ACT1t
SAT1
ACT1t
RC
TetO
ADH1t
pNIM1-RC-RDI1
3xGGGGS

Figure S7

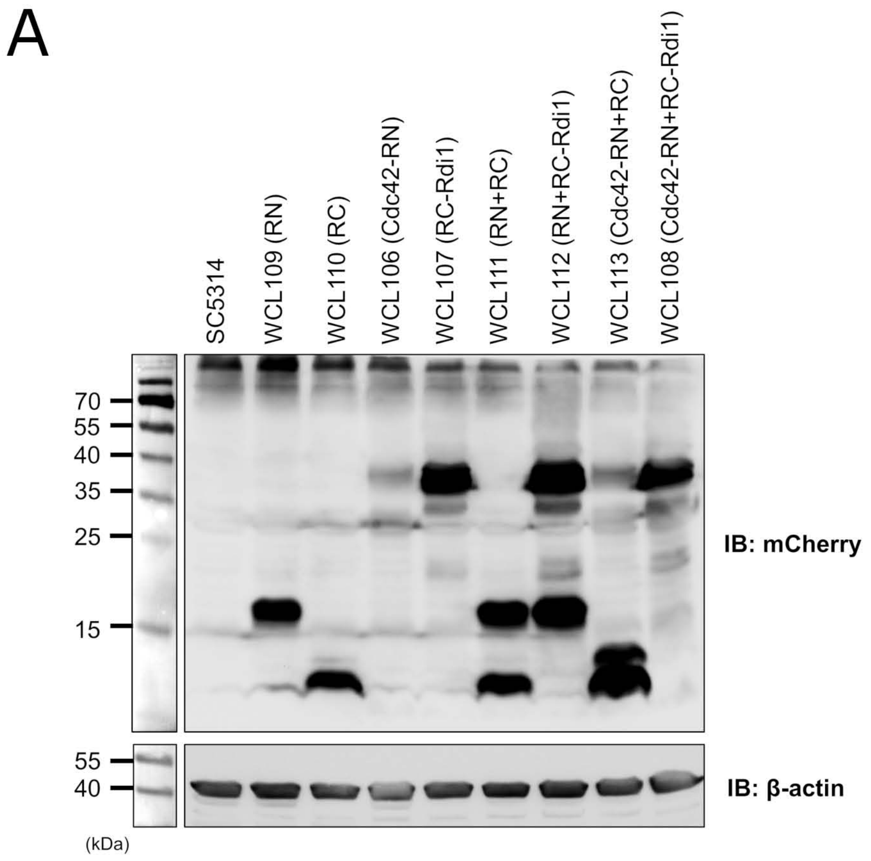
A
SC5314
WCL109 (RN)
WCL110 (RC)
WCL106 (Cdc42-RN)
WCL107 (RC-Rdi1)
WCL111 (RN+RC)
WCL112 (RN+RC-Rdi1)
WCL113 (Cdc42-RN+RC)
WCL108 (Cdc42-RN+RC-Rdi1)
70
55
40
35
25
15
IB: mCherry
55
40
IB: β-actin
(kDa)

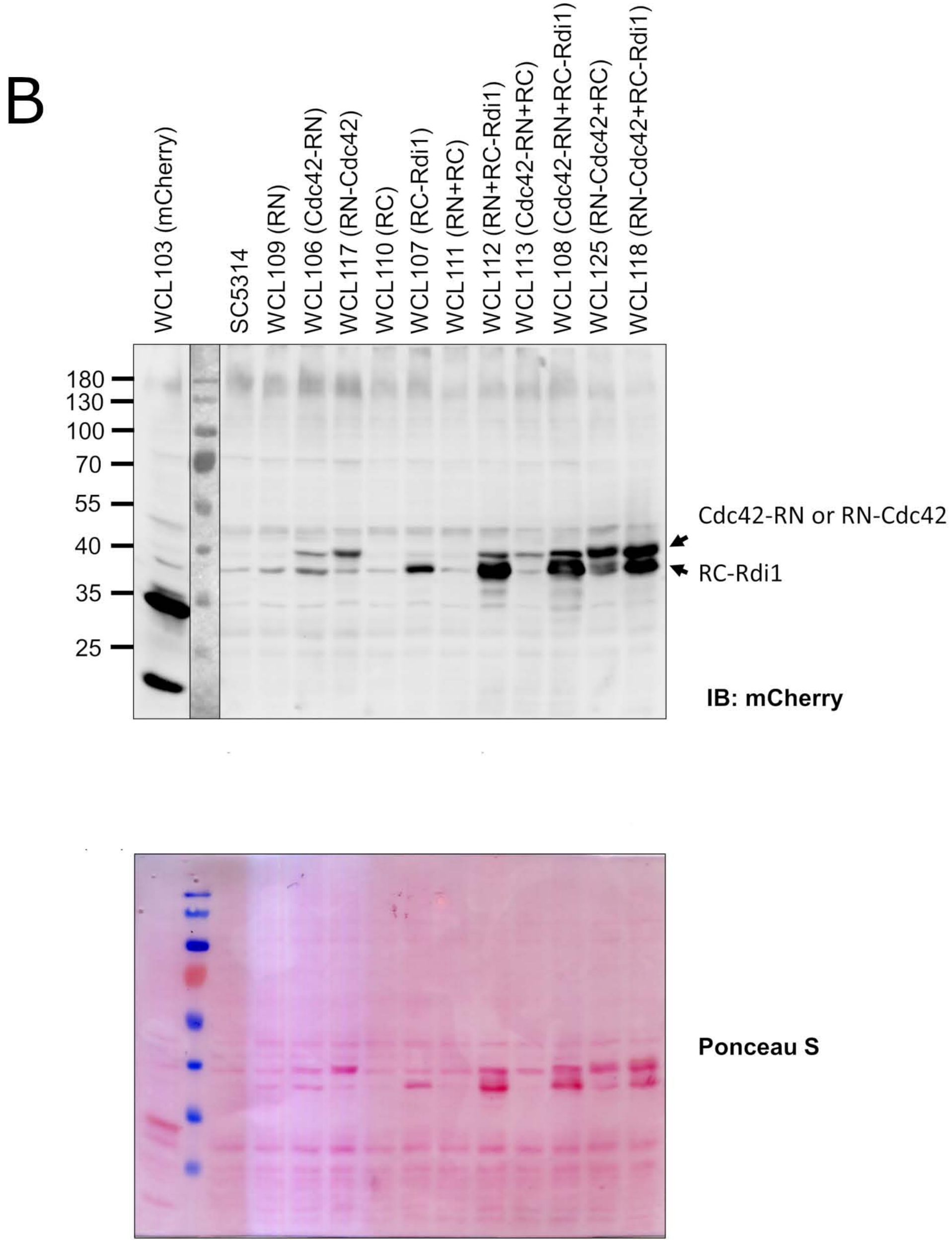
B
WCL103 (mCherry)
SC5314
WCL109 (RN)
WCL106 (Cdc42-RN)
WCL117 (RN-Cdc42)
WCL110 (RC)
WCL107 (RC-Rdi1)
WCL111 (RN+RC)
WCL112 (RN+RC-Rdi1)
WCL113 (Cdc42-RN+RC)
WCL108 (Cdc42-RN+RC-Rdi1)
WCL125 (RN-Cdc42+RC)
WCL118 (RN-Cdc42+RC-Rdi1)
180
130
100
70
55
40
35
25
Cdc42-RN or RN-Cdc42
RC-Rdi1
IB: mCherry
Ponceau S

Figure S8

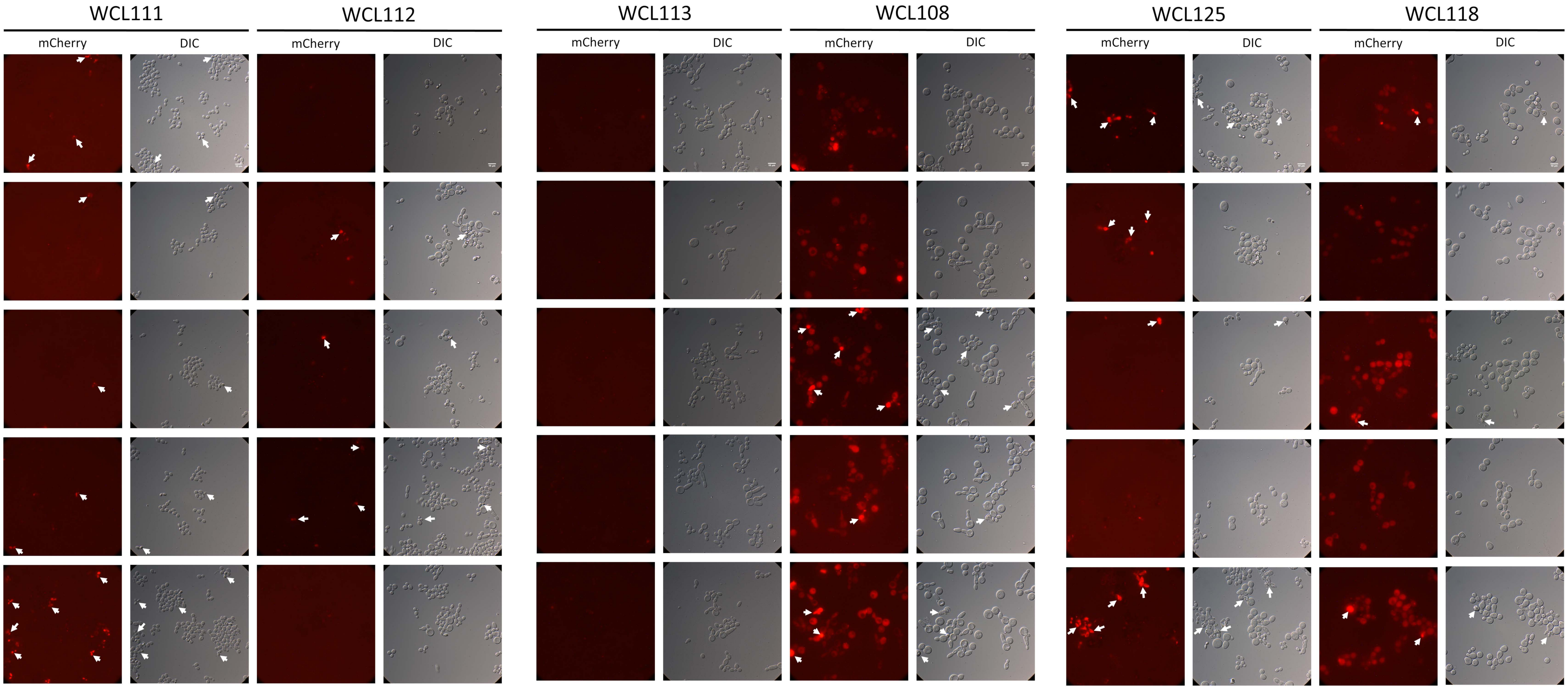

Table S1. The fold change of fluoresent proteins normalized to β-actin

The fold change of GFPmut2

	Dox	40	20	10	5	2.5	1	0.5	0.1	0
WCL101	1st	1.55269	1.09536	0.67766	0.41643	0.16501	0.00727	0	0	0
	2nd	1.21654	0.81603	0.29995	0.10638	0.01546	0	0	0	0
	3rd	1.28737	0.78532	0.36642	0.13556	0.0345	0	0	0	0
WCL102	1st	1.48509	1.17231	1.10076	0.70593	0.40464	0.11413	0	0	0
	2nd	1.26273	1.12735	0.76833	0.52711	0.21558	0.00872	0	0	0
	3rd	1.42692	1.29619	0.74946	0.36797	0.12411	0.00318	0	0	0
WCL104	1st	2.32642	1.62536	1.87504	1.54827	1.4413	1.18418	0.80493	0.12004	0
	2nd	2.85967	3.18118	3.08442	2.53326	2.24862	1.07837	0.49934	0.00199	0
	3rd	1.94842	2.85698	2.18766	1.57842	1.35224	0.45201	0.17548	0	0
WCL105	1st	2.19384	1.41578	1.49152	1.33404	0.79681	0.50129	0.29095	0	0
	2nd	1.38077	2.1934	1.70166	1.0701	0.7933	0.26362	0.10371	0	0
	3rd	1.11951	1.39998	1.54947	1.40635	0.96103	0.31902	0.0766	0	0

The fold change of mCherry

	Dox	40	20	10	5	2.5	1	0.5	0.1	0
WCL103	1st	1.3942	1.35728	1.23381	1.27457	1.05335	0.2348	0.04405	0	0
	2nd	1.84679	1.62135	1.32196	1.19537	1.05811	0.50629	0.21322	0.00564	0
	3rd	2.24408	2.88347	2.36809	2.1005	1.57599	0.48178	0.11078	0	0
WCL105	1st	1.61926	2.6041	1.88367	1.6916	1.0964	0.38805	0.13104	0	0
	2nd	3.50836	3.61307	4.83686	2.46912	1.57268	0.73033	0.35689	0	0
	3rd	2.85236	3.03205	2.5166	2.03966	1.37062	0.57371	0.18325	0	0

Table S2

Table Analyzed	WCL105				
One-way analysis of variance					
P value	< 0.0001				
P value summary	***				
Are means signif. diffe	Yes				
Number of groups	9				
F	12				
R squared	0.842				
ANOVA Table	SS	df	MS		
Treatment (between c	41	8	5.13		
Residual (within colum	7.69	18	0.427		
Total	48.7	26			
Bonferroni's Multiple C	Mean Diff	t	Significan	Summary	95% CI of diff
40 vs 20	-0.423	0.793	No	ns	-2.44 to 1.59
40 vs 10	-0.419	0.785	No	ns	-2.43 to 1.60
40 vs 5	0.593	1.11	No	ns	-1.42 to 2.61
40 vs 2.5	1.31	2.46	No	ns	-0.701 to 3.33
40 vs 1	2.1	3.93	Yes	*	0.0814 to 4.11
40 vs 0.5	2.44	4.56	Yes	**	0.422 to 4.45
40 vs 0.1	2.66	4.98	Yes	**	0.645 to 4.67
40 vs 0	2.66	4.98	Yes	**	0.645 to 4.67
20 vs 10	0.00403	0.00755	No	ns	-2.01 to 2.02
20 vs 5	1.02	1.9	No	ns	-0.998 to 3.03
20 vs 2.5	1.74	3.25	No	ns	-0.278 to 3.75
20 vs 1	2.52	4.72	Yes	**	0.504 to 4.53
20 vs 0.5	2.86	5.36	Yes	**	0.845 to 4.87
20 vs 0.1	3.08	5.78	Yes	***	1.07 to 5.10
20 vs 0	3.08	5.78	Yes	***	1.07 to 5.10

10 vs 5	1.01	1.9	No	ns	-1.00 to 3.03
10 vs 2.5	1.73	3.25	No	ns	-0.282 to 3.75
10 vs 1	2.52	4.71	Yes	**	0.500 to 4.53
10 vs 0.5	2.86	5.35	Yes	**	0.841 to 4.87
10 vs 0.1	3.08	5.77	Yes	***	1.06 to 5.09
10 vs 0	3.08	5.77	Yes	***	1.06 to 5.09
5 vs 2.5	0.72	1.35	No	ns	-1.29 to 2.73
5 vs 1	1.5	2.82	No	ns	-0.512 to 3.52
5 vs 0.5	1.84	3.45	No	ns	-0.171 to 3.86
5 vs 0.1	2.07	3.87	Yes	*	0.0522 to 4.08
5 vs 0	2.07	3.87	Yes	*	0.0522 to 4.08
2.5 vs 1	0.783	1.47	No	ns	-1.23 to 2.80
2.5 vs 0.5	1.12	2.1	No	ns	-0.892 to 3.14
2.5 vs 0.1	1.35	2.52	No	ns	-0.668 to 3.36
2.5 vs 0	1.35	2.52	No	ns	-0.668 to 3.36
1 vs 0.5	0.34	0.638	No	ns	-1.67 to 2.35
1 vs 0.1	0.564	1.06	No	ns	-1.45 to 2.58
1 vs 0	0.564	1.06	No	ns	-1.45 to 2.58
0.5 vs 0.1	0.224	0.419	No	ns	-1.79 to 2.24
0.5 vs 0	0.224	0.419	No	ns	-1.79 to 2.24
0.1 vs 0	0	0	No	ns	-2.01 to 2.01

Table S3	Fluorescence intensity	Background	Relative fluorescence intensity
111-01	403135180	269639288	133495892
111-02	448720573	443938763	4781810
111-03	555902346	506644777	49257569
111-04	559683988	481147510	78536478
111-05	594341131	343077849	251263282
112-01	680061973	379374875	300687098
112-02	528347707	466163889	62183818
112-03	617667851	131066558	486601293
112-04	519397343	510711891	8685452
112-05	466118492	339227103	126891389
113-01	634830657	382575063	252255594
113-03	447672375	288624417	159047958
113-04	599250648	411185737	188064911
113-05	506836950	383156649	123680301
113-06	543291082	315674767	227616315
108-02	605187621	66832980	538354641
108-03	764939886	62036319	702903567
108-05	583481883	138802228	444679655
108-06	689366439	54893543	634472896
108-07	712927804	23101343	689826461
125-01	579177698	271183183	307994515
125-02	403557409	177314939	226242470
125-03	532050741	69337245	462713496
125-05	393385689	221194820	172190869
125-06	547854337	148766586	399087751
118-04	706757063	94501878	612255185
118-05	690875860	65625461	625250399
118-07	737296897	53086554	684210343
118-08	720793820	31605912	689187908
118-09	622248178	95229345	527018833
